# Systematic discovery of novel eukaryotic transcriptional regulators using sequence homology independent prediction

**DOI:** 10.1186/s12864-017-3853-9

**Published:** 2017-06-26

**Authors:** Flavia Bossi, Jue Fan, Jun Xiao, Lilyana Chandra, Max Shen, Yanniv Dorone, Doris Wagner, Seung Y. Rhee

**Affiliations:** 1 Department of Plant Biology, Carnegie Institution for Science, Stanford, California, 94305 USA; 2 Department of Biology, University of Pennsylvania, Philadelphia, Pennsylvania 19104-6084 USA; 3 Department of Biology, Stanford University, Stanford, California, 94305 USA

**Keywords:** Genes with unknown function, Transcriptional regulators, Coactivators, Polycomb repressive complex 2

## Abstract

**Background:**

The molecular function of a gene is most commonly inferred by sequence similarity. Therefore, genes that lack sufficient sequence similarity to characterized genes (such as certain classes of transcriptional regulators) are difficult to classify using most function prediction algorithms and have remained uncharacterized.

**Results:**

To identify novel transcriptional regulators systematically, we used a feature-based pipeline to screen protein families of unknown function. This method predicted 43 transcriptional regulator families in *Arabidopsis thaliana*, 7 families in *Drosophila melanogaster,* and 9 families in *Homo sapiens*. Literature curation validated 12 of the predicted families to be involved in transcriptional regulation. We tested 33 out of the 195 Arabidopsis putative transcriptional regulators for their ability to activate transcription of a reporter gene *in planta* and found twelve coactivators, five of which had no prior literature support. To investigate mechanisms of action in which the predicted regulators might work, we looked for interactors of an Arabidopsis candidate that did not show transactivation activity *in planta* and found that it might work with other members of its own family and a subunit of the Polycomb Repressive Complex 2 to regulate transcription.

**Conclusions:**

Our results demonstrate the feasibility of assigning molecular function to proteins of unknown function without depending on sequence similarity. In particular, we identified novel transcriptional regulators using biological features enriched in transcription factors. The predictions reported here should accelerate the characterization of novel regulators.

**Electronic supplementary material:**

The online version of this article (doi:10.1186/s12864-017-3853-9) contains supplementary material, which is available to authorized users.

## Background

A gene product’s function can be described by its subcellular localization, the biological process in which it participates, and its molecular function (e.g. biochemical activity) using the Gene Ontology (GO) nomenclature [1]. Although these characteristics can be predicted for proteins that lack experimental data using bioinformatic tools, the molecular function of 25–75% of proteins in sequenced genomes is still unknown because they lack enough sequence similarity to characterized proteins [2–5]. Predictors that infer molecular function based on protein structure or other properties such as patterns of native disorder have been developed [6], but their application is constrained by the limited number of available protein structures and the molecular functions that display differential patterns of disorder, respectively [4, 6–8]. The inference of biological processes using algorithms that incorporate sequence-independent criteria have been performed successfully, but they are not suitable for inferring molecular function [9–12].

The uncharacterized proteins in various organisms are enriched in taxon-specific proteins that might be important for species-specific metabolism, developmental programs, or adaptation to environmental niches [13, 14]. Since these proteins lack sequence similarity to known proteins, features that are independent from sequence homology can be used to infer their molecular function. For example, transcriptional coactivators lack obvious motifs in their protein sequences [15], but have other characteristics such as nuclear localization and the ability to alter transcription of target genes, which can be used to identify new regulators within a set of uncharacterized genes. Here, we sought to predict novel transcriptional regulators by using sequence-homology independent features such as subcellular localization, biochemical properties and experimental data.

In this paper, we define transcriptional regulators as proteins that alter transcription through their direct interaction with other elements of transcription. These transcriptional regulators include DNA-binding proteins such as transcription factors (TFs) and non DNA-binding proteins such as: 1) coactivators and corepressors that bind and alter TF activity, 2) taxon-specific regulatory subunits of chromatin remodelers and modifiers, and 3) scaffold proteins that bridge the interaction between the transcriptional machinery (e.g. RNA polymerase II holoenzyme and associated factors, coactivator complexes, chromatin remodelers and modifiers) and TFs. We anticipated that the predicted proteins might have roles in transcriptional initiation, termination, or RNA processing [16, 17].

To predict novel transcriptional regulators, we built a computational pipeline that combines three features: nuclear localization, a high percentage of disordered amino acids [18, 19], and the ability to activate transcription of a reporter gene [20, 21]. We used this pipeline to screen unknown protein families that lack sequence similarity to known proteins and identified 43 novel candidate transcriptional regulator families in *Arabidopsis thaliana* (Arabidopsis), 7 in *Drosophila melanogaster* (fruit fly), and 9 in *Homo sapiens* (human). We found support for the predictions in the literature and through in silic﻿o tests.

To investigate the mechanisms of action by which the predicted regulators might act on transcription, we assessed which of the predicted candidates could act as coactivators by testing 33 Arabidopsis candidates from 25 families in an *in planta* transactivation assay. We found 12 coactivators, of which 7 had literature support for being transcriptional regulators and 5 were novel. To uncover other potential mechanisms of action, we looked for interactors of one of the candidates without transactivation activity, which was selected because a knockout mutant line showed a visible growth defect. We name this candidate as *CHIQUITA1* (*CHIQ1*). *CHIQ1* belongs to a plant-specific family of eleven members in Arabidopsis and participates in organ size determination. Biochemical characterization of protein interactions indicates that CHIQ1 might regulate transcription by interacting with other members of its family and a subunit of the Polycomb Repressive Complex 2 (PRC2). Our computational pipeline has enabled assignment of potential molecular function to 195 of ~4000 proteins of unknown function in Arabidopsis. We further showed that our pipeline could be easily implemented in other organisms.

## Results

### A feature-based computational pipeline for predicting novel transcriptional regulator families

To build a pipeline to predict novel transcriptional regulators in Arabidopsis, we explored the feasibility of using features found in some eukaryotic TFs' protein-protein interaction domains such as intrinsically disordered regions and transactivation ability because these features are also found in other transcriptional regulators. We focused on the following features: nuclear localization, a high percentage of disordered amino acids [18, 19], and the ability to activate transcription of a reporter gene in yeast (autoactivation) [20, 21]. To evaluate the selected features, we first examined whether they were in fact enriched in Arabidopsis TFs. To test whether the nuclear localization and the high percentage of disordered amino acids features were enriched in TFs, we predicted the subcellular localization and the percentage of disordered amino acids of each Arabidopsis protein and compared the average values for the TFs to those for the entire Arabidopsis proteome. Another filter was the autoactivation activity. Autoactivation refers to the ability of a protein to activate transcription in yeast when it is fused to the DNA-binding domain of the yeast TF GAL4 (GAL4BD) and in the absence of another protein fused to the activation domain of GAL4. Large - scale yeast two hybrid studies have identified proteins that have autoactivation activity [22]. The autoactivation data obtained from large-scale screenings [22] and our own interactome covers 28% of the Arabidopsis protein-encoding genes and was used to test whether the ability to activate transcription (autoactivation feature) was enriched in TFs.

**Fig. 1:**
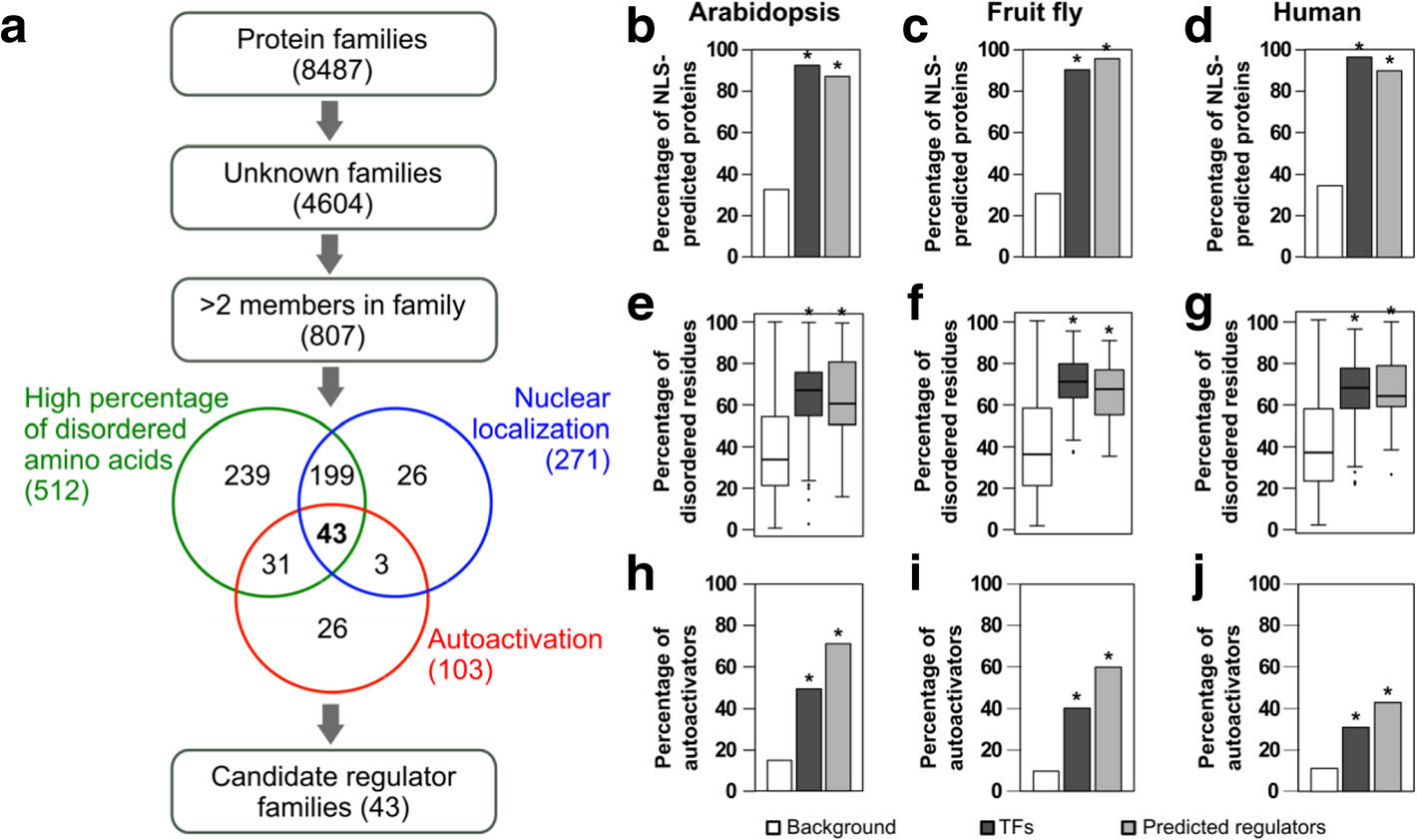
Feature-based prediction pipeline to identify novel transcriptional regulator families. **a** Pipeline work flow: First, Arabidopsis protein families were filtered based on their size and the GO annotations of their members. Then, uncharacterized families with more than 2 members were filtered based on subcellular localization patterns using Yloc [98], percentage of disordered residues using Predisorder [99], and the ability of at least one member to activate transcription of a reporter gene in yeast (autoactivation) [22, 103–107]. Numbers in the Venn diagram represent the number of families with most members being nuclear localized (blue), high percentage of disordered residues (green) and autoactivation in yeast (red). Families that met all criteria (intersection of the Venn diagram) were considered as candidate regulator families. **b**-**j** Proportion of proteins predicted to contain nuclear localization signal (NLS) (**b**-**d**), distribution of the percentage of disordered amino acid residues (**e-g**), and proportion of proteins with autoactivation activity (**h**-**j**) in the background (white), TFs (dark gray), and predicted regulators (light gray). The background corresponds to all proteins in Arabidopsis (**b**, **e**, **h**), fruit fly (**c**, **f**, **i**), or human (**d**, **g**, **j**) genomes or the set of proteins that were tested for autoactivation in yeast (**h, i, j**). * = *p*-value <0.0001, chi-square test with Yates correction (**b**-**d** and **h**-**j**) or t-test (**e**-**g**)

As expected, all three features were significantly enriched in TFs compared to all the proteins in the genome (Fig. 1b, e, h, white and dark gray bars). We then used these three features to classify Arabidopsis protein families that contain only proteins of unknown molecular function (Fig. 1a). First, we filtered the families that contained at least three members to increase the stringency of prediction based on statistical support. Then, we filtered the families based on the three TF-enriched features. Of the 807 Arabidopsis families of unknown molecular function with at least three members, 43 were enriched in all three TF-associated features and therefore were considered as putative transcriptional regulator families in Arabidopsis (Fig. 1a, b, e, h, light gray bars). These families consisted of 195 proteins (Additional file 1: Table S1). To determine the contribution of each feature towards identification of the regulators, we compared the proportion of the predicted regulator families in each filtered set versus the genome. The autoactivation activity feature contributed the most to enriching for transcriptional regulator families (Additional file 1: Table S2).

The application of the same pipeline to yeast, fruit fly, and human identified 7 candidate families (containing 23 proteins) in fruit fly and 9 families (containing 49 proteins) in human (Additional file 1: Figure S1b-c, Tables S3 and S4). No families were identified in yeast (Additional file 1: Figure S1a). The autoactivation data for yeast was obtained from [23] and the autoactivation data for fruit fly and human was obtained from the DroID and CCSB databases, respectively. The autoactivation activity was evaluated in yeast cells containing a protein of interest fused to GAL4BD. The autoactivation data obtained covers 90% of yeast, 55% of fruit fly, and 87% of human protein-encoding genes. The proportion of uncharacterized families with at least three members in Arabidopsis is 3 to 9 times greater than in yeast, fruit fly, and human (Additional file 1: Figure S2a) and filtering by family size removed most uncharacterized families in yeast, fruit fly, and human. Despite this difference, the proportion of the predicted candidate families among the uncharacterized families with at least three members is similar (4–7%) in Arabidopsis, fruit fly, and human (Additional file 1: Figure S2b). These data suggest that the thresholds defined for the three features in Arabidopsis have similar prediction power in other organisms and that the pipeline identifies fewer families in yeast, fruit fly, and human because most of the uncharacterized proteins in these organisms belong to families of 1–2 members. Therefore, constraining the predictions by family size might preclude the identification of a large number of potential transcriptional regulators in fungal and animal species. To expand our predictions (particularly in fungi and animals), we predicted regulators from families with 1–2 members using our current pipeline and found 152 regulators in Arabidopsis, 248 in yeast, 105 in fruit fly, and 200 in human.

### In silico and literature-based support of the predictions

We evaluated the performance of our pipeline using several independent approaches. First, we applied the pipeline to all protein families with molecular function annotations and at least three members and calculated precision and recall based on the number of TFs and transcriptional regulators that were predicted. Precision refers to the proportion of annotated TFs and transcriptional regulators [24] in the predicted genes, while recall refers to the fraction of all annotated TFs and transcriptional regulators belonging to ﻿families with at least three members [24] identified by the pipeline. The pipeline’s precision was 60% for Arabidopsis (Additional file 1: Figure S3a), 57% for fruit fly, and 62% for human, while recall was 58% for Arabidopsis (Additional file 1: Figure S3b), 92% for fruit fly, and 80% for human. Assessing performance at the protein level is more stringent than doing so at the family level since the prediction was made at the family level, but we chose to be more conservative in our analysis. Furthermore, we analyzed how the features contributed to precision and recall in Arabidopsis. We found that each feature can identify most of Arabidopsis TFs, but precision is low when used alone (Additional file 1: Figure S3). This is expected since the individual features are not restricted to TFs [8, 25, 26]. By using the filters in combination, we improved precision at the cost of recall (Additional file 1: Figure S3). Since we were interested in proving the concept for the approach to see if we can find novel regulators, we opted to maximize precision at the cost of recall.

We also analyzed the pipeline’s precision and recall when identifying TFs from families with 1–2 members. We found precision was 22% for Arabidopsis, 18% for yeast, 30% for fruit fly, and 17% for human, while recall was 14% for Arabidopsis, 26% for yeast, 37% for fruit fly, and 28% for human. This indicates that our current pipeline performs poorly to predict regulators in families of less than 3 members. Therefore, we did not analyze these candidate regulators further. Instead, we focused our work on the candidate regulators that belong to families of 3 or more members.

Our second approach to evaluate the pipeline’s performance was to seek for additional lines of evidence that implicate the candidate proteins in transcriptional regulation. Since GO annotations do not capture information from all literature, we performed a literature-based validation by manually curating the available literature on the candidate genes. Our criteria for calling a protein to be implicated in transcriptional regulation based on literature evidence included: 1) members belong to a characterized TF family [27–29] or coactivator complex [30]; 2) at least one member of the family affects transcriptional activity in vivo [31–33] or the activity of its TF partner by direct physical interaction [34, 35]; 3) the candidate genes contain a DNA binding domain [33, 36]; or 4) orthologs in other species have been implicated in transcriptional regulation [37–40] (e.g. EMSY-like proteins that are orthologous to human EMSY, which has been implicated in altering transcription via chromatin modification [38–43]). We found that 51 Arabidopsis proteins in 9 families and 9 fruit fly proteins in 3 families that were annotated as unknown (i.e. annotated to the root GO term molecular function [44]) when the pipeline was run, now have additional evidence in the literature that potentially implicates them in transcriptional regulation (Additional file 1: Tables S1, S3, and S4). To date, none of the human candidate families have been associated with transcriptional regulation in the literature. To assess the pipeline for false positive predictions, we looked for literature evidence that indicated that the candidates are involved in functions other than transcriptional regulation. None of the Arabidopsis and fruit fly candidates had such evidence. Members of one human candidate family are membrane channels, suggesting they might be false positive predictions [45]. In addition, two human families were considered as potentially false positive because they have experimental evidence that indicates localization to compartments such as Golgi and the cornified envelope (differentiated plasma membrane of keracinocytes), not currently known to be sites of transcriptional regulation [46–48]. Based on literature curation, 9 families in Arabidopsis and 3 in fruit fly were considered true positive and 3 families in human were considered false positive predictions. This analysis indicated that we identified 34 putative novel transcriptional regulator families in Arabidopsis, 4 in fruit fly, and 6 in human.

Third, certain amino acids are overrepresented in transcriptional activation domains such as acidic, glutamine-rich, and proline-rich activation domains [49]. Moreover, yeast transcription factors are enriched in asparagine, glutamine, serine, proline, and aspartic acid [23]. To test if the predicted transcriptional regulators are also enriched in these amino acids, we analyzed the maximal number of these amino acids in 20-amino ac﻿i﻿d sliding win﻿dow﻿s per protein in the whole genome, annotated TFs, and the predicted regulators. We also included glutamic acid and the number of acidic amino acids (glutamic and aspartic acid) in this analysis. Similarly to yeast [23], the amino acid sequences of the Arabidopsis and fruit fly TFs are enriched in all six amino acids individually and in acidic amino acids (Additional file 1: Figure S4a and b, black bars), while the human TFs are enriched in five of the six amino acids and in the amount of acidic amino acids (Additional file 1: Figure S4c, black bars). The Arabidopsis candidate regulators are enriched in all six amino acids: aspartic acid (Bonferroni-corrected *p*-value: 2.43E-13, t-test), glutamic acid (Bonferroni-corrected *p*-value: 2.83E-05, t-test), asparagine (Bonferroni-corrected *p*-value: 0.013, t-test), glutamine (Bonferroni-corrected *p*-value: 3.12E-09, t-test), serine (Bonferroni-corrected *p*-value: 1.37E-13, t-test), and proline (Bonferroni-corrected *p*-value: 1.99E-05, t-test) and acidic amino acids (Bonferroni-corrected *p*-value: 3.28E-10, t-test) (Additional file 1: Figure S4a). Similarly, the human candidates were significantly enriched in aspartic acid (Bonferroni-corrected *p*-value: 0.031, t-test), glutamic acid (Bonferroni-corrected *p*-value: 1.38E-05, t-test), serine (Bonferroni-corrected *p*-value: 6.94E-06, t-test), and acidic amino acids (Bonferroni-corrected *p*-value: 6.07E-08, t-test) (Additional file 1: Figure S4c). Although the fruit fly candidates contained a similar number of these amino acids as the TFs, only aspartic acid (Bonferroni-corrected *p*-value: 0.039, t-test) and the sum of acidic amino acids (Bonferroni-corrected *p*-value: 0.041, t-test) were significantly enriched (Additional file 1: Figure S4b). The lack of statistical significance for the other amino acids in fruit fly could be a result of a smaller sample size.

Finally, we evaluated the performance of our pipeline by analyzing the interactors of the predicted transcriptional regulators. Since the predicted proteins lack known DNA binding domains, they might be recruited to target promoters through other proteins. Consistent with this hypothesis, we found that 2﻿8﻿ Arabidopsis candidates, 7 fruit fly candidates, and 7 human candidates physically interact with proteins implicated in transcription, including TFs, chromatin remodeling, and histone modifying complexes (Additional file 1: Table S5) in yeast-two-hybrid or co-immunoprecipitation studies. However, proteins implicated in transcriptional regulation were not enriched among the candidates’ interactors, perhaps due to the small sample size. Among the candidates that interact with transcription-associated proteins, 20 Arabidopsis and one fruit fly candidates had literature support for being involved in transcriptional regulation. The predicted regulators interact more commonly with TFs and﻿ transcriptional regulators (TRs) [24] than chromatin remodeling and histone modifying proteins (Additional file 1: Table S5). In fact, ~37% of Arabidopsis regulator candidates with protein-protein interaction data interact with a TF/TRs. This value is significantly higher (fold-change = 1.7, *p*-value = 0.0051, Fisher-test) from what is observed for the Arabidopsis proteome, where 2﻿2% of all proteins with protein interaction data interact with a TF.

### The predicted transcriptional regulator families are taxon-specific

**Fig. 2:**
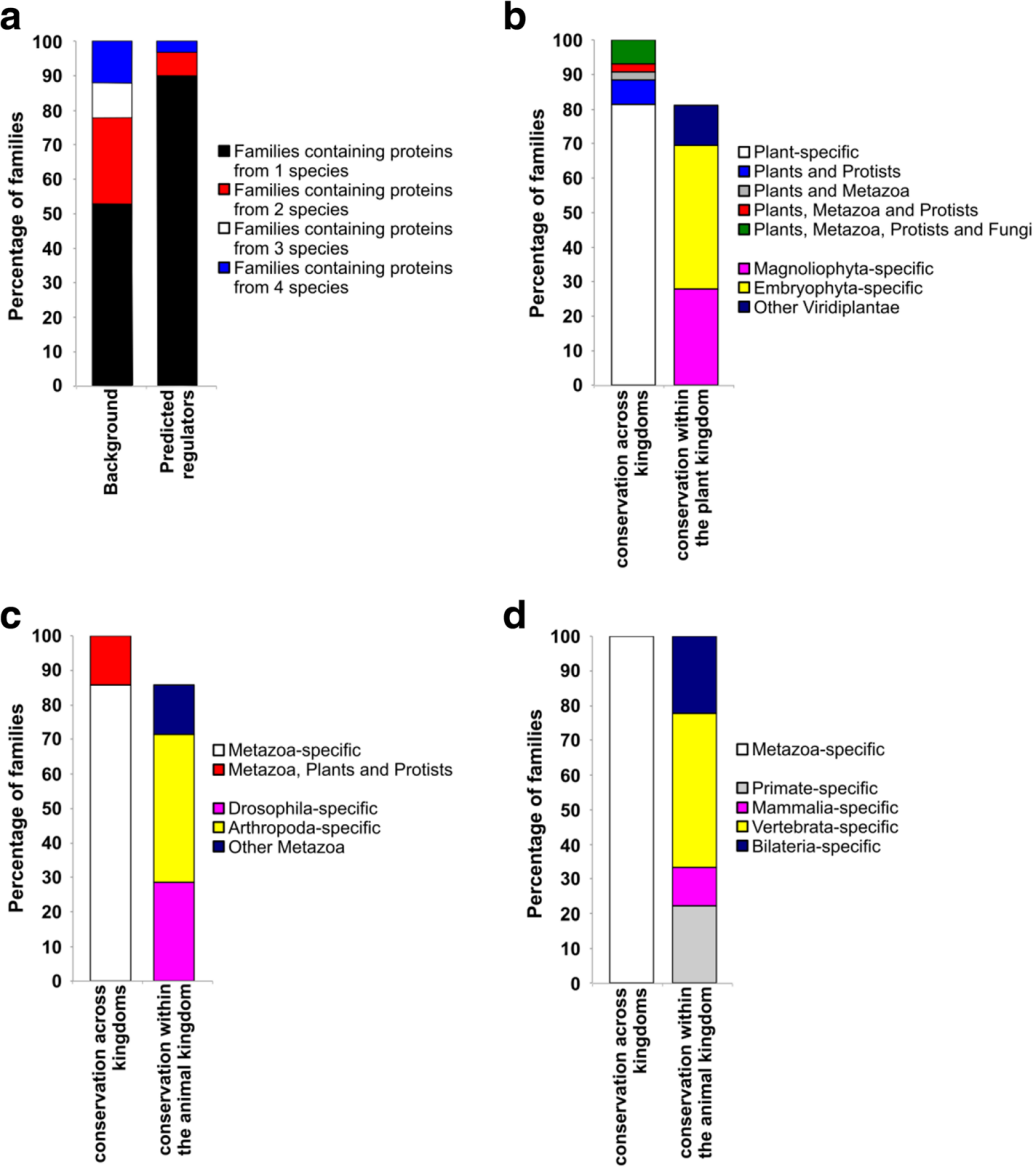
Ortholog distribution of the predicted regulator families. **a** Proportion of families that contain proteins from one, two, three or four species in all the families and in the predicted transcriptional regulator families generated by OrthoMCL [109]. **b**-**d** Ortholog distribution of the predicted regulator families in Arabidopsis (**b**), fruit fly (**c**), and human (**d**) using data from Ensembl Genomes [50] to classify taxon specificity of the candidate families within each taxonomic domain

The predicted regulators are not similar in sequence to known genes; therefore we posited that they would not be widely conserved. To test this hypothesis, we performed two analyses. First, we combined all the proteins in Arabidopsis, yeast, fruit fly, and human to generate meta-genome protein families and ran the pipeline on the unknown families with more than two members. We identified 59 candidate transcriptional regulator families and found that most of them (~90%) contain proteins from only one species (Fig. 2a). Second, we characterized taxon-specificity of the predictions by looking for orthologs of the candidate transcriptional regulators in the Ensembl Genomes database [50] and found that 82% of Arabidopsis candidate families are conserved only within the plant kingdom (Fig. 2b). Of these, 12% have orthologs in green algae, 42% have orthologs in early land plants, and 28% have orthologs only in flowering plants. Fruit fly candidates are conserved mainly in arthropods (Fig. 2c) and human candidates are conserved mainly in vertebrates (Fig. 2d). These independent lines of evidence indicate that the predicted regulators are not widely conserved, which reinforces the use of homology-independent features for their identification and supports the notion that they might control the expression of genes involved in more taxon-specific processes or constitute components of taxon-specific complexes. Our results are consistent with previous reports indicating that transcription-associated proteins are generally taxon-specific [51–53].

### *In planta* analysis of transactivation ability of Arabidopsis predictions

**Fig. 3:**
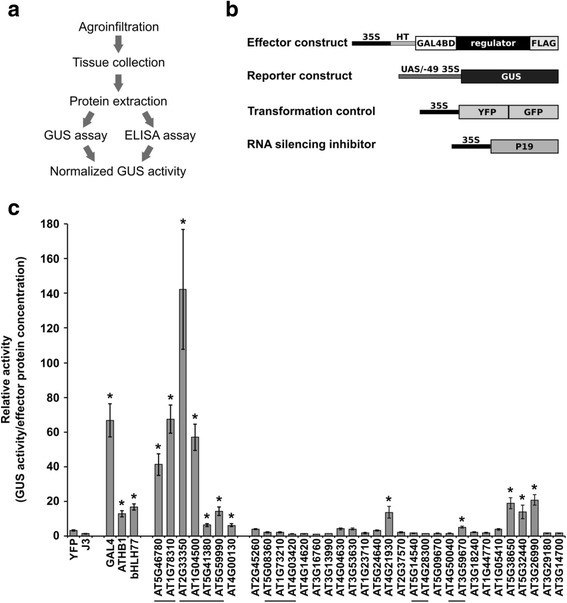
Experimental analysis of the predictions. **a** Steps of the *in planta* transactivation assay procedure from bacterial growth to quantification of the normalized transactivation activity. **b** Constructs used in the transactivation assay. **c** Average relative transactivation activity calculated as the GUS activity (nmol of 4MU/min/mg total protein) divided by the concentration of the effector protein (ng/ml). Error bars represent standard error from 3 independent experiments. The asterisk (*) indicates that the relative activity is statistically different from the YFP control (*p*-value <0.002, t-test). A line under the gene names indicates that they belong to the same family

The predicted transcriptional regulators may participate in transcription via different mechanisms. As a start, we focused on testing for transcriptional activation using Arabidopsis candidates. To determine which of the predicted Arabidopsis candidates are activators *in planta*, we developed a quantitative *in planta* transactivation assay and tested 33 Arabidopsis candidate genes from 25 families for their ability to activate transcription of a reporter gene. To select these families for *in planta* tests, we randomly selected 22 of 34 families that do not have supporting evidence for a role in transcriptional regulation and 3 of 9 families with literature evidence implicating them in transcriptional activation. From these 25 families we then selected the genes with autoactivation activity ([22] and Methods) for testing *in planta*, which resulted in 33 candidate genes. The selected candidate regulators were fused to GAL4BD (DNA binding domain that binds to the Upstream Activating Sequence (UAS) motif) and tested for their ability to activate transcription of a β-glucuronidase (GUS)-encoding reporter gene driven by a UAS-containing promoter in tobacco leaves (Fig. 3). Tobacco leaves were co-infiltrated with Agrobacterium cultures carrying four constructs (Fig. 3b): 1) the reporter construct containing the coding region of the GUS gene driven by a promoter with three copies of the GAL4 binding site (UAS) [54] and the −49 bp minimal region of the constitutive promoter 35S [55, 56]; 2) the effector construct carrying the candidate gene fused to the DNA binding domain of GAL4; 3) the fluorescent protein (YFP-GFP), which served as a transformation control; and 4) the P19 gene which suppresses RNA silencing [57]. The relative activity of each effector construct was calculated as the effector’s fluorometric GUS activity divided by its protein concentration.

We tested three negative controls: the fluorescent protein YFP and the chaperone J3 [58] fused to GAL4BD, and YFP without the GAL4BD domain (Fig. 3c and Additional file 1: Figure S5). GAL4BD-YFP showed higher background GUS activity compared to GAL4BD-J3 (Fig. 3c), and therefore was selected for the statistical comparison between the GUS activity detected in leaves infiltrated with the positive controls and candidate genes. The three positive controls, yeast TF GAL4, plant TFs *Arabidopsis thaliana* HOMEOBOX 1 (ATHB1) [59], and BASIC HELIX-LOOP-HELIX PROTEIN 077 (bHLH077), all acted as activators (Fig. 3c). The yeast protein GAL4 was the strongest of the positive controls, and the two plant TFs increased the expression of the reporter gene by 4–5 fold over the GAL4BD-YFP negative control (Fig. 3c).

Twelve candidate genes in eight families showed transcriptional activity *in planta* (Fig. 3c). All 7 candidates from 3 families with literature support showed transcriptional activity. Of the 26 candidates from 22 families with no other supporting information, we identified 5 novel activators that belong to 5 unknown families (Fig. 3c and Additional file 1: Table S1). The remaining candidates might be false positive predictions or proteins whose transcriptional activity depends on context such as the availability of condition- or tissue-specific interactors.

### A candidate regulator, CHIQ1, is involved in organ size determination and interacts with the Polycomb repressive complex 2 (PRC2) subunit EMF2 via the CHIQ1 family protein CHIQUITA LIKE6 (CHIQL6)

**Fig. 4:**
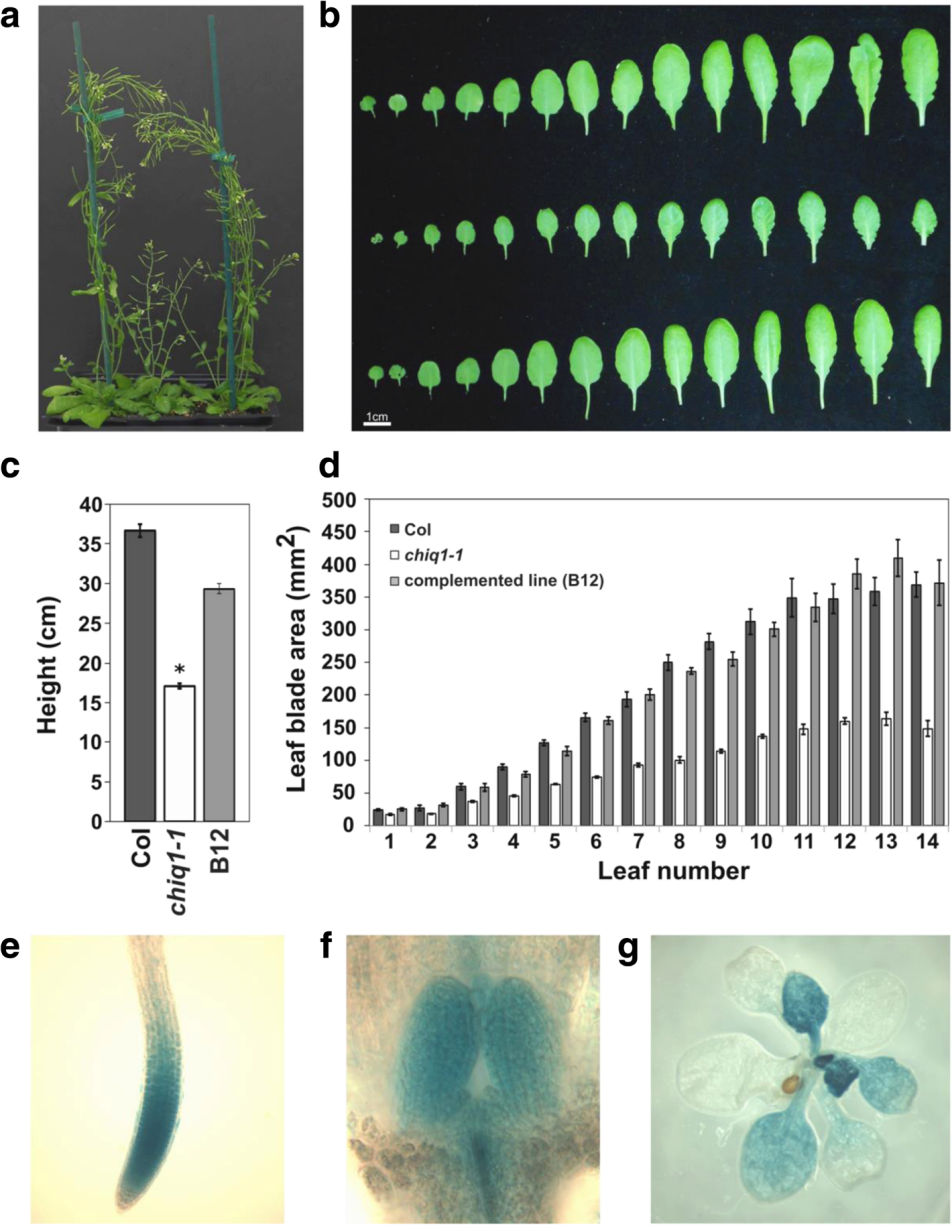
Mutants lacking *CHIQ1* have smaller organs. **a**-**b**, Whole plants (**a**) or rosette leaves (**b**), of wild type (Col-0, left or top), *chiq1–1* (middle), and *chiq1–1* complemented with *CHIQ1* (B12, right or bottom) grown in soil for 7 weeks. Leaves are ordered from the oldest (left) to the youngest (right). **c** Height of the primary inflorescence stem in wild type (black), *chiq1–1* (white), and complemented (gray) plants grown in soil for 11 weeks. Stature of *chiq1–1* plants is reduced by 53% compared to the wild type and 42% compared to the complemented line (* = *p*-value: 2E-34 against wild type and 2E-25 against complemented line, t-test). *n* = 30 per genotype from 8 independent experiments. **d** Measurements of leaf area from wild type (black), *chiq1–1* (white), and complemented (gray) plants grown in soil for 7 weeks. *n* = 8 per genotype from 3 independent experiments. **c**-**d** Error bars represent standard error from 3 independent experiments. **e**-**g** Expression of the *CHIQ1-GUS* transgene driven by *CHIQ1* promoter in the root apical meristem (**e**), shoot apical meristem and leaf primordia of 2 day-old seedlings (**f**) and rosette of 14 day-old plants (**g**) grown on MS media. Each image is a representative of at least three independent experiments with *n* = 10 plants. At least three independent transgenic lines were analyzed

Some of the predicted transcriptional regulators might act as coactivators (Fig. 3c) and others might participate in transcription through other mechanisms. To investigate other potential mechanisms by which the predicted regulators might work, we looked for interactors using proteomics. To select which genes to study, we examined mutant lines of the candidate genes that lacked transactivation activity *in planta* (Fig. 3c) for visible growth, developmental, or morphological phenotypes as many transcriptional regulators discovered through forward genetics have strong visible phenotypes [60–63]. We tested 9 homozygous insertional mutant lines of 7 candidate genes for developmental phenotypes and identified one mutant line with a severe growth phenotype (Additional file 1: Table S1). We found that plants harboring a knockout mutation in a candidate gene (TAIR: AT2G45260), named hereafter as *CHIQUITA 1 (CHIQ1*), was defective in organ size (Fig. 4). Adult plants carrying a knockout allele of *CHIQ1* (*chiq1–1*) were shorter in stature and had smaller rosette leaves, indicating that *CHIQ1* is involved in determining organ size (Fig. 4). These phenotypes were recessive and segregated as a single Mendelian locus (Additional file 1: Table S6). To confirm that a mutation in the AT2G45260 locus (*CHIQ1*) causes the small size phenotype, we introgressed the mutant allele into wild type to remove unrelated non-linked mutations and performed a linkage analysis, which indicated that the homozygous mutant allele co-segregated with the small organ size phenotype (Additional file 1: Table S7). To rule out the possibility that the phenotype is caused by a locus linked to AT2G45260, we introduced the coding region of *CHIQ1* into the homozygous mutant line. This complemented the organ size phenotype (Fig. 4), indicating *CHIQ1* is responsible for the organ size phenotype.

To study the effect of *CHIQ1* on organ size, we examined leaf size reduction in *chiq1–1* compared to the wild type. We found that size reduction varies from 28 to 60% depending on the final leaf size, with the older leaves that have smaller final leaf size decreasing less than the younger leaves that have larger final size (Fig. 4b and d). Organ shape, flowering time, and the number of leaves in the rosette were not affected in the *chiq1–1* mutant (Fig. 4b and Additional file 1: Figure S6), indicating that organ morphology and developmental transitions are independent of *CHIQ1* function. Consistent with *CHIQ1*’s potential role in leaf growth, *CHIQ1* is expressed specifically in dividing and expanding tissues (Fig. 4e-g).

**Fig. 5:**
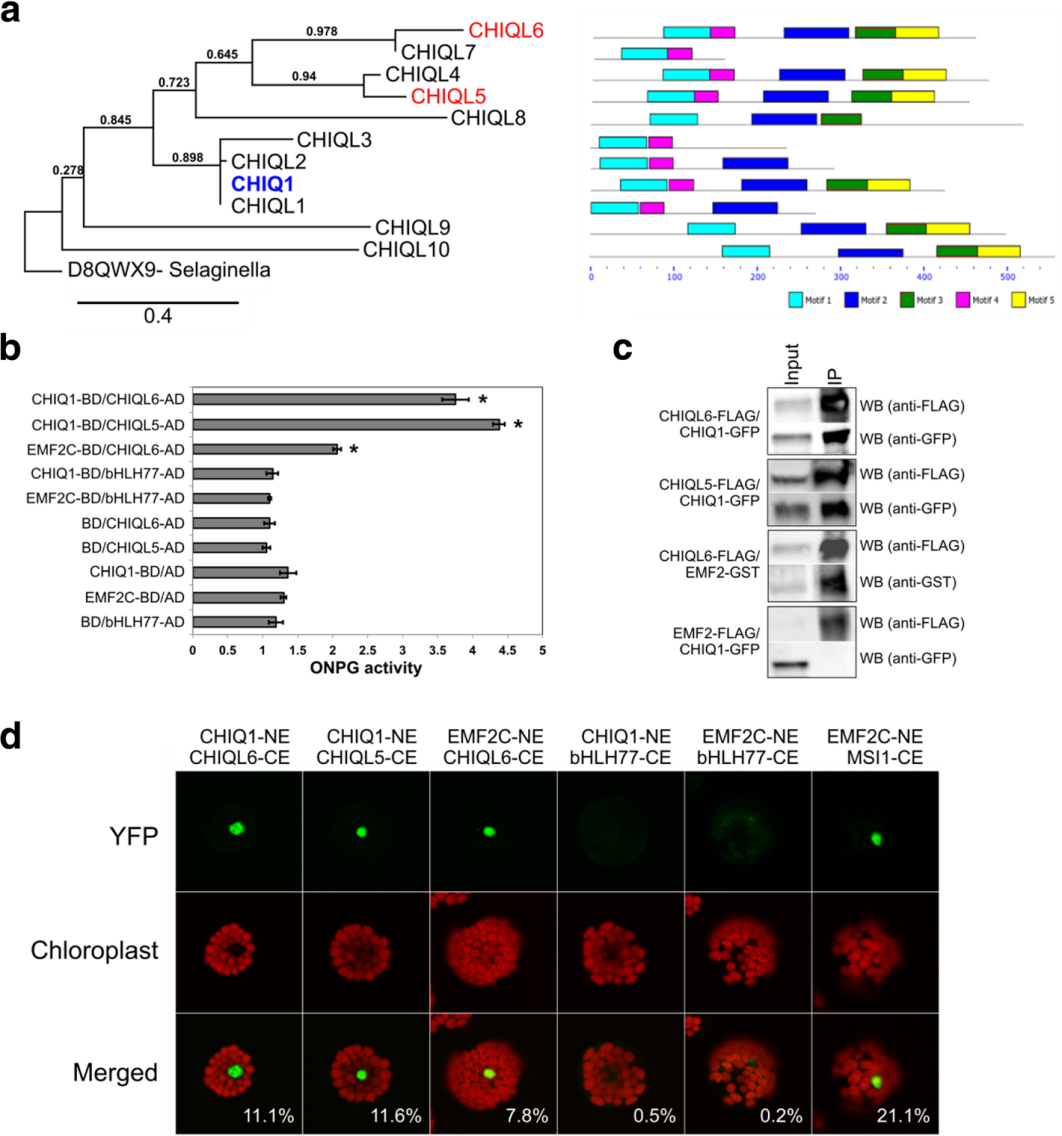
CHIQ1 family interacts with EMF2. **a** Phylogenetic tree of Arabidopsis CHIQ1 family (left) made using Phylogeny.fr [121] and motif conservation in CHIQ1 protein family (right) predicted by MEME [122]. Motifs 1 and 4 correspond to the DUF641 domain. Height of the domains indicates the degree of conservation, where taller domains are more conserved than shorter ones. CHIQ1 is in blue and CHIQ1’s interactors in red. CHIQL6 (TAIR: AT1G29300), CHIQL7 (TAIR: AT2G32130), CHIQL4 (TAIR: AT3G14870), CHIQL5 (TAIR: AT1G53380), CHIQL8 (TAIR: AT2G30380), CHIQL3 (TAIR: AT4G36100), CHIQL2 (TAIR: AT4G33320), CHIQ1 (TAIR: AT2G45260), CHIQL1 (TAIR: AT4G34080), CHIQL9 (TAIR: AT3G60680), CHIQL10 (TAIR: AT5G58960). **b**-**d** Physical interaction between CHIQ1, CHIQL6, CHIQL5, and EMF2 based on yeast two-hybrid assays (**b**), pull-down assays in tobacco (**c**), and bimolecular fluorescence complementation assays in Arabidopsis protoplasts (**d**). Pull-down assays were performed with anti-FLAG antibody and we used anti-GFP antibody to detect CHIQ1, anti-GST antibody to detect EMF2, and anti-FLAG antibody to detect CHIQL6, CHIQL5, and EMF2 in the eluted immuno-precipitate. The input corresponds to the total protein extract and IP is the eluted immuno-precipitate. Error bars in (**b**) represent standard error. * = *p*-value <0.001, t-test. In (**d**), green indicates fluorescence from reconstituted Venus fluorescent protein. Red indicates autofluorescence from the chloroplast. The percentage corresponds to the fraction of cells expressing Venus in each sample. Representative images from three independent experiments are shown (*n* = 258–321 cells per pair per experiment)

The CHIQ1 protein did not activate transcription of the reporter gene *in planta* (Fig. 3c, AT2G45260). *CHIQ1* is expressed in growing tissue and since the *in planta* transactivation assay is performed in mature tissue, some key CHIQ1 interactors important for function might be missing. Alternatively, CHIQ1 might be a scaffold protein without any transcriptional activity on its own. CHIQ1 belongs to a plant-specific family of eleven members that lack a known DNA binding domain and contain the domain of unknown function 641 (DUF641) (Fig. 5a, DUF641 corresponds to motif 1 and 4).

To gain insight into the mode of action of *CHIQ1,* we immuno-purified CHIQ1::GFP interactors. Based on LC/MS-MS, 201 proteins were associated specifically with CHIQ1::GFP compared with the GFP control, including four members of the CHIQ1 family, EMBRYONIC FLOWER 2 (EMF2), a subunit of PRC2, two proteins (PICKLE-RELATED1 (PKR1) and PICKLE-RELATED2 (PKR2﻿)) that belong to the CHROMODOMAIN-HELICASE-DNA-BINDING PROTEIN (CHD) chromatin remodeling family, and several TFs (Additional file 1: Table S8). Orthogonal protein-protein interaction tests between CHIQ1 and eleven potential interactors confirmed direct interactions between CHIQ1 and two members of CHIQ1 family: CHIQUITA1-LIKE 6 (CHIQL6) and CHIQUITA1-LIKE 5 (CHIQL5) in yeast and *in planta* (Fig. 5). An all-by-all physical interaction analysis among nine CHIQ proteins indicated that CHIQ1 also interacts with CHIQUITA1-LIKE 7 (CHIQL7) in yeast (Additional file 1: Table S9). CHIQ1 did not interact with the identified TFs in yeast-two-hybrid or pull-down assays (Additional file 1: Table S8). The interactions between CHIQ1 and PKR1 and PKR2 have not yet been tested. We confirmed the physical interaction between the PRC2 complex component EMBRIONIC FLOWER2 (EMF2) and CHIQL6 by quantitative yeast two-hybrid tests, bimolecular fluorescence complementation (BiFC) assays in Arabidopsis protoplasts, and pull-down assays in tobacco (Fig. 5b-d). Based on these results, we tested the physical interactions between nine members of the CHIQ1 family and the core subunits of the PRC2 complex using yeast-two-hybrid assays. Besides CHIQL6, two members of the CHIQ1 family, CHIQL7 and CHIQUITA1-LIKE 10 (CHIQL10), interacted with the methyltransferases CURLY LEAF (CLF) and SWINGER (SWN) (Additional file 1: Table S5). The role of these interactions in transcriptional regulation remains to be elucidated.

## Discussion

### A feature-based pipeline can identify novel transcriptional regulators in eukaryotes

Advances in genome sequencing revealed numerous protein-encoding genes that are unknown for function. Sequence similarity to characterized genes has been the main paradigm for predicting gene function. Under this paradigm, many genes whose sequences are not similar to known genes remain recalcitrant to function prediction. To discover new types of transcriptional regulators from genes whose sequences are not similar to known genes, we developed a computational pipeline that leverages genome-wide information and the corpus of knowledge gathered about transcriptional regulators. Using this pipeline on Arabidopsis, fruit fly, and human genomes, we predicted 34, 4, and 6 novel protein families with potential roles in taxon-specific transcriptional regulation. The pipeline’s performance evaluated by its ability to identify known TFs and by literature and in silico data indicated that we were able to predict regulators with good precision and recall. Furthermore, experimental tests in Arabidopsis identified 5 novel activator families and one family that might regulate transcription via histone modification. Since we did not perform any experimental tests on the animal candidates, it is currently unknown whether they act as transcriptional regulators in their corresponding native environments and what their potential mechanisms are. Overall, our pipeline found a substantial number of potentially new transcriptional regulators in Arabidopsis, fruit fly, and human, which opens the door for new hypotheses and axes of investigation.

We sought to identify novel transcriptional regulators, in particular proteins that have potential activation or repression activities without necessarily binding directly to DNA. Since these types of proteins do not have obvious, conserved domains in their protein sequence, we used criteria that are independent of sequence homology. To select those criteria, we focused on features found in TFs’ protein-protein interaction domains such as high degree of disorder and autoactivation because they are shared with other regulators. These features acted additively to predict regulators with good precision and recall (Additional file 1: Figure S3). For example, the autoactivation feature did not perform well when used alone (Additional file 1: Figure S3, red bar). Several reasons might have contributed to this: 1) incomplete data for the autoactivation activity of proteins in yeast; 2) some activator domains (i.e. proline- and glutamine-rich domains) have variable autoactivation activity in yeast [21, 64–69]; and 3) proteins with no transcriptional regulatory function can alter the expression of the reporter gene in yeast. Despite the limited predictive power of the autoactivation feature, when it is used in combination with the other features, it increased their combined precision (Additional file 1: Figure S3a). These results show that combining different types of characteristics improves the predictive power of the pipeline.

To our knowledge, this is the first report describing a filter-based pipeline that incorporates TF-features to find novel transcriptional regulators in genomes. Further improvements to this pipeline include the addition of features that were identified in the in silico validation in this study (e.g. amino acid composition, taxon-specificity, and physical interaction with TFs).

### Five novel Arabidopsis transcriptional regulators might be coactivators

Our pipeline was designed to identify transcriptional regulators: novel TFs and other regulatory proteins that can alter gene expression directly by physical interaction with transcription-associated proteins such as chromatin remodelers and modifiers, TFs, and components of the general transcriptional machinery. Therefore, we anticipated that the predicted regulators might work through different mechanisms. Some non-DNA binding regulators might be coactivators or corepressors that alter TF binding or activity, while others might be scaffold or regulatory subunits of coactivator complexes and chromatin remodelers and modifiers.

*In planta* transactivation assays have been used to test the effect of TFs and other regulatory proteins on transcription [56, 59, 70, 71] indicating that the transactivation activity of transcriptional regulators can be assessed successfully using this system. To identify which predicted Arabidopsis regulators activate transcription *in planta* when tethered to the promoter of a reporter gene, we developed a transient transactivation assay. Using this system, we identified 5 novel coactivators. The remaining tested candidates that did not show activator activity could be false positives, repressors or unsuitable for testing in our current system. Transcriptional activators usually work combinatorially [72] and the individual contribution of certain activators to transcription is context-dependent [73]. For example, the activator activity might depend on posttranslational modifications or the interaction with specific partners that are lacking in our system. Transactivation activity could also depend on promoter context [74] since many TFs depend on the position of the TF binding motifs within the promoter to activate transcription [75–77]. It is also possible that some of the predicted regulators act as repressors in plants. This is supported by the fact that some of the predicted candidates with literature support (such as members of the LOB and OVATE [31, 78] and EMSY [39, 40] families) function as repressors, indicating that our pipeline could identify repressors. Since we predicted at the family level, we might identify repressors simply because they belong to a candidate family whose members include both activators and repressors as is the case for some TF families [71, 79]. Alternatively, some of the candidates with activator activity in yeast might be repressors in plants, depending on the repertoire of available interacting proteins. This dual transcriptional function has been observed for some TFs [73, 80–83]. Finally, the predicted regulators might work as regulatory subunits or scaffold proteins of the transcriptional machinery, in which case determining their partners of interaction will give us insight into their mechanism of action. While we tested three positive (TFs) and two negative (nuclear-localized, non-transcriptional regulators) controls in this system, a wider selection of proteins (e.g. selected randomly from the genome) to test for transcriptional activity in this system would provide a better baseline to assess the recovery rate of transcriptional activators. Further characterization of the candidates that show transcriptional activity would be required to confirm their roles in transcriptional regulation.

### Novel regulators from CHIQ1 family might control gene expression through Polycomb repressive complex 2 (PRC2)

To investigate CHIQ1’s mechanism of action, we searched for interactors involved in transcriptional regulation using proteomics, yeast-two-hybrid, and BiFC assays and found that CHIQ1 directly interacts with other CHIQ1 family members and, via CHIQL6, interacts with the EMF2 subunit of the repressive complex PRC2.

The PRC2 is a transcriptional repressor complex that silences genes by tri-methylating lysine 27 of histone H3 (H3K27me3) in the nucleosomes of target loci [84]. The complex consists of four widely conserved core subunits: 1) a methyltransferase; 2) a zinc finger and VEFS domain-containing protein that provides stability; 3) a WD40 repeat protein that binds to H3K27me3; and 4) a WD40 repeat protein that binds nucleosomes [84]. The Arabidopsis PRC2 complex exists in three variants, named after the zinc finger/VEFS domain protein, EMF2-PRC2, VRN2-PRC2 and FIS2-PRC2 [85]. PRC2 is essential during developmental transitions in plants [86]. In Arabidopsis, FIS2-PRC2 participates in embryogenesis, EMF2-PRC2 is involved in cell fate determination and cell differentiation in leaves and in the transition from vegetative to reproductive development, and VRN2-EMF2 is important for the transition to flowering after a cold period [86].

Based on our protein-protein interaction data, we hypothesize that CHIQ1, CHIQL6, and EMF2-PRC2 might work together to modulate transcription. The precise roles of CHIQ proteins in transcriptional regulation and how they affect EMF2-PRC2 function remain to be elucidated. PRC2 can be regulated by controlling its recruitment to target genomic regions or its enzymatic activity. PRC2 core subunits do not have sequence-specificity; therefore PRC2 target-specificity relies on its interaction with long non-coding RNAs, transcription factors, or other histone modifications to recognize target sites [87–91]. Overall, molecular mechanisms for PRC2 recruitment are poorly understood [92, 93]. Moreover, little is known about how PRC2’s activity can be modulated. One possible role for CHIQ proteins might be to work as adaptors that link chromatin regulators with elements that control sequence specific recruitment or have a regulatory role on the methyltransferase activity of PRC2.

## Conclusions

This paper describes a “reverse genomics” approach that systematically identifies previously uncharacterized transcriptional regulators, which might control the activity of TFs or chromatin regulators. For example, the discovery of CHIQ1 family and its initial characterization identified a novel plant-specific family that might work with PRC2 complex during growth. We hypothesize that the predicted regulators might form higher order complexes with TFs and chromatin modifying complexes to fine-tune transcriptional activity. Other, more unexpected mechanisms of transcriptional regulation could also be revealed in these candidate genes. Our approach contributes to assigning a molecular function to previously unknown genes, which still represent 25–75% of genes in eukaryotic genomes [3–5], and accelerating the discovery of new regulators of transcription.

## Methods

### Clustering Arabidopsis proteins in families based on overall sequence similarity

*Arabidopsis thaliana* (Arabidopsis) protein sequences were downloaded from Phytozome (ftp://ftp.jgi-psf.org/pub/compgen/phytozome/v9.0/Athaliana/annotation/Athaliana_167_protein_primaryTranscriptOnly.fa.gz) [94]. The proteins were clustered based on sequence similarity (BLASTP e-value cut-off of 1E-5) using BLAST+ 2.2.29 and the Markov Cluster Algorithm (MCL version 12–068) [95] at four different inflation values: 1.4, 2, 4, and 6. Results from the different clustering schemes were consistent with each other, with the inflation value of 1.4 being the least restrictive and generating larger clusters and 6 being the most restrictive and generating smaller clusters. To determine the most appropriate inflation value externally, we randomly picked seven transcription factor families (CCAAT-DR1, C2C2-YABBY, G2-like, GeBP, NAC, bZIP, and MYB) with 2, 6, 16, 40, 73, 96, and 131 members from the Arabidopsis Transcription Factor Database (http://arabidopsis.med.ohio-state.edu/AtTFDB/) [96] as the gold standard clusters. To measure clustering quality, we used Jaccard Index between the MCL-generated clusters and the gold standard clusters. Inflation value 4 was chosen for subsequent analysis because it had the highest overlap with gold standard data (average Jaccard Index of 0.71) among the four inflation values.

### Selection of functionally unknown clusters

To select the clusters containing only the functionally unknown proteins, the Gene Ontology (GO) annotations [44] for molecular function were extracted from the GO annotation file in The Arabidopsis Information Resource (TAIR) [97] website (ftp://ftp.arabidopsis.org/home/tair/Ontologies/Gene_Ontology/ATH_GO_GOSLIM.txt, downloaded 09/03/2013). Proteins annotated to the root molecular function term (GO:0003674) as well as proteins without any molecular function GO annotation were considered as unknown.

### Selection of candidate transcriptional regulator families

The regulator families were predicted from the set of unknown families with more than two members. An unknown cluster was considered to be a candidate transcriptional regulator family if it met the following three criteria: 1) more than 50% of its members were predicted to localize to the nucleus; 2) the average ratio of disordered amino acid residues of its members was higher than 0.34﻿1 (a cutoff set for the 5% cumulative distribution of all transcription factors); and 3) at least one of its members was able to activate transcription in yeast based on experimental evidence (autoactivation). The subcellular localization of Arabidopsis proteins was predicted using YLoc [98]. The disordered amino acids in each protein were predicted using Predisorder 1.1 [99]. We calculated the ratio of disordered residues for each protein as the number of disordered amino acids divided by the protein length. Predisorder does not work for proteins larger than 2500 amino acids. To overcome this restriction, long proteins were split into pieces for prediction. To address the potential bias in the prediction from the split ends, two splitting scenarios were implemented. In the first method, the sequence of long proteins was split equally into n parts, where n is the minimal number for each piece shorter than 2500 amino acids. In the second method, the sequence was split equally into (n + 1) parts. Predisorder was applied to predict the disordered amino acids for each piece resulting from the two splitting methods. The ratio of disordered residues of the long protein was then calculated as the average ratio of disordered residues of the pieces from the two splitting methods. The list of proteins with autoactivation activity was obtained from The Arabidopsis Interactome Mapping Consortium [22] and in-house tests (see Autoactivation assays in yeast b﻿elow). The autoactivation data covers ~28% of Arabidopsis proteome and refers to the ability of Arabidopsis proteins to activate transcription of a reporter gene in yeast when fused to the DNA binding domain of the yeast transcription factor GAL4. This construct is used in yeast-two-hybrid assays that study protein-protein interaction and its transcriptional activity must be evaluated before performing yeast-two-hybrid studies, as an important negative control. We asked The Arabidopsis Interactome Mapping Consortium for their results of the aforementioned negative control.

### Application of the pipeline to other organisms

The pipeline, as described for Arabidopsis, was applied to three model organisms: yeast, fruit fly, and human. Yeast protein sequences were downloaded from Ensembl (version R64–1-1). Fruit fly protein sequences were downloaded from Ensembl (BDGP5) and the longest transcript for each gene was used. The human complete proteome (filtered for ‘reviewed’) was downloaded from Uniprot on 07/24/2014. GO annotation files of the three organisms were downloaded from Gene Ontology Consortium (geneontology.org, [44]) (SGD [100] on 7/26/2014, FlyBase [101] on 7/15/2014, and EBI GO Annotations *Homo sapiens* [102] on 7/10/2014). The yeast proteins that activate transcription in yeast were obtained from [23]. The fruit fly proteins with autoactivation activity were provided by Dr. Russ Finley (personal communications, DroiD database) from previously published high throughput protein-protein interaction studies [23, 103–107] and the human proteins with autoactivation activity was provided by Dr. Tong Hao (personal communications, Dana-Farber Cancer Institute, USA). The autoactivation data covers ~90%, 55% and 87% of the yeast, fruit fly, and human proteome, and the rationale for obtaining and using the data has already been explained in the section “Selection of candidate transcriptional regulator families”.

### Fold-enrichment analysis

To determine the fold enrichment each criterion yielded towards identification of the regulators, we counted the number of families that passed each criterion in the genome. We then calculated the fold enrichment of each criterion using a hypergeometric test. The fold enrichment was obtained by dividing the ratio of the predicted regulator families within the families that met each criterion by the ratio of the predicted regulator families in all the families in the genome ﻿f﻿or all criteria except autoactivation and final candidates. To calculate fold enrichment for the autoac﻿tivation criterion and final candidates, only the families with at least one member tested for autoactivation was used in the d﻿enominator ratio.

### Amino acid composition and protein-protein interaction analyses

We counted the number of each amino acid found in 20 amino acid sliding windows with an overlap size of 19 amino acids as described in [23] for all Arabidopsis, fruit fly,﻿ and human proteins and compared the averages of the maximum of each amino acid in the predicted candidate proteins, TFs, and the whole proteome using a t-test. *P*-values were adjusted using Bonferroni correction.

To find interactors of the predicted regulators and calculate the percentage of proteins that interact with TFs/TRs, we used the file: "BIOGRID-ORGANISM-Arabidopsis_thaliana_Columbia-3.4.149.tab2" from the BioGRID website (https://thebiogrid.org/download.php)﻿ [108]. To calculate the percentage of proteins that interact with TFs/TRs, we divided the number of proteins that interact with a TF/TR by the number of proteins that have﻿ any interaction data. To calculate the percentage of predicted regulators that interact with a TF/TR, we divided the number of predicted regulators that interact with a TF/TR by the number of predicted regulators that have any interaction data.

### Taxon specificity analysis of candidate genes

All proteins from Arabidopsis, yeast, fruit fly and human genomes were combined and clustered by OthoMCL [109]. To be consistent with the single-genome clustering method described in the prediction pipeline, inflation value 4 was chosen for MCL clustering of the meta-genomes. To estimate the degree of taxon specificity of the predicted regulators, the protein families were grouped into four categories containing proteins from 1, 2, 3, and 4 species. The percentage of candidate regulators in each category was calculated. To find orthologs of the predicted regulators in other organisms, we manually extracted the ortholog information for each gene from Ensembl Genomes database [50].

### Construction of plasmids

For the *in planta* transactivation assay, we constructed the reporter vector 3xUAS/−49 35S:GUS and 38 effector vectors. To construct the vector 3xUAS/−49 35S:GUS, two complementary primers containing three Upstream Activating Sequence (UAS) [110] *cis*-elements and the -49 bp region of the constitutive tobacco promoter 35S (3xUAS-4935S_for: 5′ GGGGACAAGTTTGTACAAAAAAGCAGGCTTCCGGCCGCGGAGGACTGTCCTCCGTGCACGGAGGACTGTCCTCCGATCGGAGGACTGTCCTCCGTGCAATCCTTCGCAAGACCCTTCCTCTATATAAGGAAGTTCATTTCATTTGGAGAGGAGGCGCGCCGACCCAGCTTTCTTGTACAAAGTGGTCCCC 3′ and 3xUAS-4935S_rev: 5′ GGGGACCACTTTGTACAAGAAAGCTGGGTCGGCGCGCCTCCTCTCCAAATGAAATGAACTTCCTTATATAGAGGAAGGGTCTTGCGAAGGATTGCACGGAGGACAGTCCTCCGATCGGAGGACAGTCCTCCGTGCACGGAGGACAGTCCTCCGCGGCCGGAAGCCTGCTTTTTTGTACAAACTTGTCCCC 3′) were annealed at 70 °C for 10 min, cooled down on ice, cloned directly into pDONR221, and transferred into the binary vector pGWB633 [111] using Gateway cloning (Life Technologies). To construct the backbone of the effector vector, we modified the binary vector pB7HFC3_0 (donated by Dr. Dmitri Nusinow, Donald Danforth Plant Science Center, USA) to create pHT-GAL4BD-HFC by: 1) cloning the DNA binding domain of GAL4 amplified from the plasmid pDEST32 (Life Technologies) at the SpeI restriction site (located between the end of the 35S promoter and the beginning of the left Gateway cloning cassette of pB7HFC3_0) and 2) cloning the HT leader sequence amplified from pEAQ-HT-DEST1 [112] between the end of the 35S promoter and the beginning of the GAL4 DNA binding domain. The HT leader sequence was cloned using megaprimers generated by PCR using the following primers: HT-for: 5′ CTATTCTAGTCGACCTGCAGGCGGCCGCTATTAAAATCTTAATAGGTTTTG 3′, and HT-rev: 5′ CTTGTTCGATAGAAGACAGTAGCTTCATACTAGTGTTTGATCGAATTTGGGCAG 3′; and the QuickChange II XL Site-directed mutagenesis protocol (Agilent Technologies). To generate pB7HFC3_0, the vector pB7HFC [113] was used as template to amplify two overlapping fragments using primers pDAN0193 5′–TGCCCGCCTGATGAATGCTC–3′ and pDAN0239 5′–GTGATGCGATCCTCCTCCCACTTTGTACAAGAAAGCTGA–3′ to generate attR2A, and pDAN0240 5′–TCAGCTTTCTTGTACAAAGTGGGAGGAGGATCGCATCAC–3′ and pDAN0223 5′–ATTCTCATGTATGATAATTCGAGG–3′ to generate attR2B. The PCR products attR2A and attR2B were diluted, mixed and re-amplified with primers pDAN0193 and pDAN0223 to generate the fragment attR2C. The vector pB7HFC was linearized by digestion with EcoRI and XbaI (NEB) and recombined with attR2C fragment using In-Fusion® HD cloning (Clontech) to generate the pB7HFC_3.0 vector, which was verified by sequencing before further use. Entry vectors for the following genes AT2G45260, AT1G23710, AT4G21930, AT4G14620, AT1G73210, AT4G03420, AT4G04630, AT5G08360, AT3G16760, AT5G24640, AT5G14540, AT5G09670, AT3G18240, AT1G44770, AT1G05410, AT5G38650, AT3G14700, AT3G59670, AT5G32440, AT3G26990, AT3G53630, AT2G37570, AT3G50040, AT3G23690 (*bHLH077*) and AT3G44110 (*J3*) were constructed as follows: the coding sequence of each gene was amplified by PCR from pUNI51 vectors acquired from the Arabidopsis Biological Resource Center (ABRC) or from genomic DNA, and cloned into the entry vector pENTR-SD or pDONR221 (Life Technologies). The entry vector containing the activation domain of *GAL4* was amplified by PCR from pDEST22 and cloned into pENTR-SD (Life Technologies). The entry vectors containing the genes AT3G29180, AT3G13990, AT4G28300, AT5G46780, AT1G78310, AT2G33350, AT1G04500, AT5G41380, AT5G59990 and AT4G00130 were obtained from ABRC. Dr. Enrico Magnani provided the entry vector containing the *ATHB1* gene (INRA, Centre de Versailles-Grignon, France) and Dr. Zhiyong Wang donated the entry vector containing the gene *YFP* (Carnegie Institution for Science, USA). These 38 genes were transferred from the entry vectors into the binary vector pDB-HT-GAL4-HFC, using Gateway cloning (Life Technologies), to create the effector vectors.

To construct a binary vector that overexpresses the recombinant gene CHIQ1-GFP, the AT2G45260 (CHIQ1) protein-coding sequence was amplified by PCR from Col-0 genomic DNA, cloned into the entry vector pENTR-SD (Life Technologies), and transferred to the binary vector pGWB5 [114], using Gateway cloning (Life Technologies), to create the vector pGWB5-CHIQ1.

To construct the translational fusion CHIQ1-GUS, 642 bp of the promoter region (including the 5′ UTR) plus the coding region of AT2G45260 lacking the stop codon was amplified by PCR from Col-0 genomic DNA, cloned into pENTR-SD (Life Technologies), and transferred into pGWB3 [114] using Gateway cloning (Life Technologies) to create the vector pGWB3-CHIQ1.

To construct the vectors for the yeast two-hybrid assays, we amplified the following genes: AT4G33320, AT4G34080, AT2G32130, AT5G58960, AT1G29300, AT1G53380, AT3G14870, AT5G60680, AT3G23690 (*bHLH077*), AT1G18040 (*CDKD1;3*), AT1G76010, AT5G65630 (*GTE7*), AT3g20740 (*FIE*), AT5G58230 (*MSI1*), AT5G51230 (*EMF2*, full length protein and C-terminal [115]), AT4G16845 (*VRN2*, full length protein and C-terminal [115]), AT2G23380 (*CLF*, N-terminal region lacking the SET domain [115]), AT4G02020 (*SWN*, N-terminal region lacking the SET domain [115]) from genomic DNA, cDNA, or plasmids obtained from ABRC. The PCR products were cloned in the entry vector pENTR-SD or pDONR221 and transferred to the yeast destination vectors pDEST22 and pDEST32, using Gateway cloning (Life Technologies). The entry vector containing the gene AT5G28540 (U16271) was obtained from ABRC. AT5G45050 (*WRKY16*) in pDEST22 was donated by John Gierer and Dr. Todd Mockler (Donald Danforth Plant Science Center, USA).

The vectors for the BiFC and pull-down assays were constructed by transferring the entry clones of AT2G45260, AT1G29300, AT1G53380, AT3G23690, AT5G58230, and AT5G51230 described above into the following plant destination vectors: pUC-SPV-NE^GW^, pUC-SPV-CE^GW^, pB7HFC3_0, pGWB5 [114], and pGWB24 [114]. The vectors pUC-SPV-NE^GW^ and pUC-SPV-CE^GW^ were modified from the pDEST-VYNE/CE(R)^GW^ vectors [116] by switching the split Venus-Gateway cassette into pUC18 backbone.

### Autoactivation assays in yeast

We tested the following genes: AT1G04500, AT1G05040, AT1G05730, AT1G15600, AT1G15610, AT1G15620, AT1G15630, AT1G15640, AT1G17400, AT1G22980, AT1G44010, AT1G50690, AT1G54180, AT1G72490, AT2G15590, AT2G20590, AT2G24140, AT2G29880, AT2G32050, AT2G33350, AT2G33400, AT2G36540, AT2G36550, AT2G38823, AT2G45260, AT3G01015, AT3G02125, AT3G54520, AT3G54530, AT4G00390, AT4G27660, AT4G30830, AT4G30830, AT5G41380, AT5G59990, AT4G00130. The entry vectors in pDONR221 were obtained from ABRC (except for AT2G45260, whose cloning was described in the previous section) and were transferred to the yeast destination vector pDEST32 using Gateway cloning (Life Technologies). Genes in the pDEST32 vector (Life Technologies) plus the vector pEXP502 (Life Technologies) were co-transformed into the yeast strain MaV203 (Life Technologies) following the manufacturer’s instructions. The transformation reaction was plated on selective media (6.7 g/L Yeast nitrogen media without amino acids (DIFCO) supplemented with 2% glucose (SIGMA), 2% agar (Carolina), 1X leucine (Clontech), and 1X tryptophan (Clontech) at 30 °C for 3–5 days. Positive colonies were tested for β-galactosidase activity on nylon membranes as described in the ProQuest manual (Life Technologies).

### Plant material and growth conditions

*Nicotiana benthamiana* plants were grown in soil (PRO-MIX® HP Mycorrhizae) for 5–6 weeks at ﻿22°C in 16/8 photoperio﻿d. *Arabidopsis thaliana* plants were grown at 22 °C in 16/8 photoperiod either in soil (PRO-MIX® HP Mycorrhizae) or in 0.5X Murashige and Skoog basal salt mixture (MS) media (PhytoTechnologies Laboratories) (pH 5.7), supplemented with 0.8% agar (Difco) and 1% sucrose (SIGMA). Seeds were stratified in the cold room (~4 °C) for four nights to break dormancy.

We obtained *Arabidopsis thaliana* ecotype Col-0 (wild type) plants and 9 mutant lines (in Col-0 background) from ABRC (stock numbers included in Additional file 1: Table S1). We generated plants overexpressing AT2G45260 by introducing the transgene 35Spro:CHIQ1-GFP from the plasmid pGWB5-CHIQ1 into the *chiq1–1* mutant background (ABRC stock number: SALK_064001). Five transgenic lines were selected in 1X MS medium (PhytoTechnologies Laboratories), supplemented with 1% sucrose (Sigma-Aldrich) and 50 mg/L kanamycin (Gibco). Two homozygous lines (A13 and B12) were used for the macroscopic phenotypic characterization and the line B12 was used for the scanning electron microscopic and immunoprecipitation studies.

Transgenic lines carrying the translational fusion CHIQ1-GUS were generated by introducing the transgene *CHIQ1pro:CHIQ1-GUS* from the plasmid pGWB3-CHIQ1 into Col-0 plants. The expression of the transgene in seedlings was analyzed in at least six independent lines.

### Linkage analysis and functional complementation of *chiq1–1* mutant phenotype

The Arabidopsis mutant line SALK_064001 (*chiq1–1*) was backcrossed to Col-0 once. The resulting F_1_ plants were selfed, and 125 F_2_ seeds were planted in soil. After seven weeks, the stature of each plant was scored as either short or tall (wild type), and the genotype of 59 plants was assessed by PCR. The seeds from one of the backcrossed homozygous lines (line 22) were used for all the phenotypic analyses.

The *chiq1–1* plants were transformed with the transgene *35Spro:CHIQ1-GFP*. F_1_ heterozygous plants were planted on soil and their stature was scored after seven weeks. The line B12 was chosen for further phenotypic studies, including organ size and developmental traits (see below). B12 was selfed and homozygous plants were selected using kanamycin.

### Phenotypic analyses

#### Plant height

The height of the primary inflorescence stem was measured with a ruler from plants grown in soil for 11 weeks. At least 30 individuals per genotype (Col-0, *chiq1–1*, line B12) from eight independent experiments were measured.

#### Flowering time

The number of leaves with a visible petiole was counted daily from day 16 after sowing to day 39–40 in soil-grown plants. To determine bolting time, the number of days that passed between sowing and when the inflorescence of at least 1 cm in height appeared was counted. These experiments were performed eight times (*n* = 9–12 per genotype per experiment).

#### Leaf size

Leaf size was measured from plants grown in soil for 7 weeks. Fully expanded rosette leaves with a visible petiole were scanned and their blade area was measured with ImageJ. This experiment was performed three times and eight individuals from each genotype were analyzed.

In all cases, we performed t-tests to determine statistical significance.

### *In planta* transactivation assay

Fully expanded 3rd, 4th, or 5th leaves from 5 to 6 week-old tobacco (*Nicotiana benthamiana*) plants were co-infiltrated with the reporter construct, the effector construct, a construct overexpressing a fluorescent marker (transformation control) and another overexpressing the protein P19 [57]. Agrobacterium cultures carrying each construct were grown overnight at 28 °C. Each culture was washed four times in infiltration buffer (10 mM MgCl_2_ (omniPur, EMD), 10 mM MES (pH 5.6) (J. T. Baker) and 100uM acetosyringone (Sigma-Aldrich)) and diluted to reach an OD_600_ of 0.8. The effector and reporter construct were infiltrated at a ratio of 9 to 1. Each combination was infiltrated in one leaf (four ~1 cm-diameter dots per leaf) from different plants [117]. We used the transcription factors GAL4, ATHB1 [59], and bHLH077 as positive controls, and YFP and the chaperone J3 [58] as negative controls. Three days after infiltration, leaves (two per plasmid combination) with similar GFP expression were collected. T﻿he four infiltrated areas in each leaf were excised and pooled into one sample. We performed 3 independent infiltrations per plasmid combination resulting in 6 samples per gene.

Protein extracts were prepared and used for GUS enzymatic activity measurements and ELISA assays. Total protein content was extracted using the following buffer (GUS extraction buffer: 50 mM NaHPO4 (pH 7.0) (Sigma-Aldrich), 10 mM β-mercaptoethanol (Sigma-Aldrich), 10 mM EDTA (Sigma-Aldrich), 0.1% (*w*/*v*) sodium lauryl sarcosine (Sigma-Aldrich), 0.1% (*w*/*v*) Triton X-100 (Sigma-Aldrich), and one tablet of cOmplete ULTRA protease inhibitor cocktail per 15 ml of buffer (Roche)). Protein concentration was measured using the Bradford assay (Bio-Rad). To measure the GUS enzymatic activity, 100 μg of each protein extract in GUS extraction buffer was incubated with GUS assay solution (2 mM 4-Methylumbelliferyl β-D-Glucuronide (Gold Biotechnology) in GUS extraction buffer) in a 1 ml reaction at 37 °C; and 100 ul (of this 1 ml reaction) were transferred to 1.9 ml of 0.2 M carbonate (Na_2_CO_3_) stop solution at the following time points: 0, 30, 60, 90, and 120 min. The GUS activity was measured using the Dyna Quant 200 fluorometer (Hoefer), which was blanked with 2 ml of 0.2 M carbonate solution and calibrated with 50 nM 4-MU (7-hydroxy-4-methylcoumarin, Sigma-Aldrich) solution in 0.2 M carbonate solution. The GUS activity values were calculated following the mathematical formula from the Technical Bulletin MB-470 associated with the β-glucuronidase (GUS) fluorescent reporter gene activity detection kit (Sigma-Aldrich). The effector protein concentration was measured using ELISA assays (Abcam). All effector proteins were fused to the FLAG tag (as the effector vector contains a FLAG tag in frame at the C-terminal), which enabled the use of anti-FLAG antibodies. ELISA assays were performed as follows: 20 μg of total protein was diluted in 50 mM bicarbonate/carbonate buffer (Sigma-Aldrich) and incubated overnight on a Microcolon high-binding 96-well plate (Greiner) at 4 °C, the plate was blocked with 200 ul of Immunoassay blocking (BSA free) solution (Abcam) for ~4 h at room temperature, incubated with 100 ul of 1:3000 anti-FLAG antibody (F3165, Sigma-Aldrich) overnight at 4 °C, and finally incubated with 100 ul of 1:5000 anti-mouse antibody (Santa Cruz) for ~2 h at room temperature. To read the plate: 75ul of 1-Step TM ultra TBS-ELISA substrate (Thermo Scientific) was added to each well and incubated for 30 min at room temperature, the reaction was stopped with 75 ul of stop solution (Thermo Scientific), and measured at 450 nm using a plate reader. To calculate the absolute values of concentration, we included a standard curve using FLAG peptide (Sigma-Aldrich) at the following concentrations: 0, 50, 100, 200, 300, 500, 1000, 2000, 3000, 4000, and 5000 ng/ml. All standards and samples were analyzed in triplicates (ELISA assay). The normalized GUS enzymatic activity was calculated by dividing the GUS activity (nmol of 4MU/min/mg total protein) by the concentration of effector protein (ng/ml).

### Histochemical analysis of Arabidopsis transgenic lines

Expression of the *CHIQ1-GUS* transgene driven by its native promoter was analyzed in 2 and 14 day-old seedlings grown in MS agar media. Seedlings were stained in GUS staining solution [118] at 37 °C overnight, and were destained in 70% ethanol at room temperature for 24 h. Pictures were taken with the Nikon Eclipse microscope and Leica MZ6 stereo microscope.

### Co-immunoprecipitation and mass spectrometry analysis (co-IP/MS)

Seedlings overexpressing GFP or the translational fusion CHIQ1-GFP were grown for 2 days in 0.5X MS agar media. A total of 20 g of tissue per genotype was frozen in liquid nitrogen. Tissue was ground using liquid nitrogen, and total protein content was extracted using a native buffer (100 mM sodium phosphate, pH 8.0 (Sigma-Aldrich), 150 mM sodium chloride (EMD Chemicals Inc), 5 mM EDTA (Sigma-Aldrich), 5 mM EGTA, 0.05% Triton X-100 (Sigma-Aldrich), and one tablet of cOmplete ULTRA protease inhibitor cocktail per 10 ml buffer (Roche)). Protein concentration of each extract was measured using the Bradford assay (Bio-Rad). Twenty micrograms of the polyclonal anti-GFP antibody (donated by Dr. Z. Wang, Carnegie Institution for Science, USA) were coupled to 40 μl of protein A/G magnetic beads (Thermo Scientific) following the manufacturer’s instructions. 110 mg of total protein was incubated with the antibody-coupled beads for 1.5 h at 4 °C with gentle rotation. Beads were washed four times with the extraction buffer and the protein complexes were eluted with 2X Laemmli buffer (Bio-Rad). The immunoprecipitation was verified by Western blot analysis. The eluate was run in a 4–20% gradient SDS-PAGE gel (Bio-Rad) and stained with Coomassie Brilliant Blue (Bio-Rad). Each lane in the gel was cut into seven pieces and each piece was analyzed individually using Mass Spectrometry. Samples were sent to the Vincent Coates Foundation Mass Spectrometry Laboratory (Stanford University Mass Spectrometry), where they digested the proteins with trypsin, separated the peptides using Liquid Chromatography (Waters Nano Acquity), and identified the peptides using Mass Spectrometry (LTQ-Orbitrap Velos). Data was acquired in a data dependent acquisition (DDA) mode where the top 12 most intense precursor ions were isolated and fragmented using the ion trap. Raw data was analyzed against Arabidopsis proteome extracted from NCBI (ftp://ftp.ncbi.nlm.nih.gov/genomes/refseq/plant/Arabidopsis_thaliana/, downloaded on 05/20/2014) using Sequest (Thermo Finnigan) and visualized using Scaffold (Proteome Software, Inc.). Based on decoy analysis, the false discovery rate for protein identification was set to 1% and only the proteins with a minimum of three peptides were considered in the analysis. Proteins pulled down in the negative control samples (i.e. GFP overexpressing plants) and CHIQ1 overexpressing plants were compared, and only the proteins present in the samples from CHIQ1 overexpressing plants were considered as possible interactors of CHIQ1 (Additional file 1: Table S8). We selected candidate interactors for further testing if they belonged to the CHIQ1 family or had a transcription-related GO annotation.

### Yeast two-hybrid assays

PRC2 components (AT3g20740 (FIE), AT5G58230 (MSI1), AT5G51230 (EMF2), AT4G16845 (VRN2), AT2G23380 (CLF), and AT4G02020 (SWN)) in pDEST32 were transformed into yeast strain AH109 using the Frozen-EZ yeast transformation II kit according to the manual (ZYMO research). Nine proteins from CHIQ1’s family (AT4G33320, AT4G34080, AT2G45260, AT3G60680, AT3G14870, AT1G29300, AT1G53380, AT2G32130, and AT5G58960) in pDEST22 were transformed into yeast strain Y187. To obtain double transformants, single haploid colonies from each transformation were grown overnight, each pair mixed, and incubated for one day at 28 °C. To select for the colonies that contain both pDEST22 and pDEST32 constructs, the resulting diploid cells were plated onto selective media without leucine and tryptophan. To screen for interacting pairs, three colonies of each combination were streaked onto selective media without leucine, tryptophan, and histidine (SC-Leu-Trp-His) supplemented with or without 3-amino-1,2,4-triazole. As a background control, empty pDEST22 was paired with all histone modifiers and chromatin remodelers in pDEST32 and empty pDEST32 was paired with all candidate genes in pDEST22. Interaction pairs that grew better or faster in all three colonies compared with the background controls were considered as positive.

We further used the ortho-nitrophenyl-β-D-galactopyranoside (ONPG) assay to quantify their interaction. To perform the ONPG assay of β-galactosidase activity in yeast, we grew nine independent colonies of each positive pair in pools of three (i.e. three colonies per sample) overnight in 2 ml of selective media without leucine and tryptophan at 28–30 °C. Yeast cells were precipitated and resuspended in Z buffer (40 mM sodium phosphate monobasic monohydrate, 60 mM sodium phosphate dibasic heptahydrate, 10 mM potassium chloride, and 1 mM magnesium sulfate heptahydrate). Enzymatic reaction was performed in 500 μl of Z-buffer plus 50 μl of 0.1% SDS, 50 μl of chloroform, and 100 μl ONPG (4 mg/ml) at 37 °C for 2–30 min. The reaction was stopped with 1 M sodium carbonate. The enzymatic activity was measured at OD_420_, and the units of β-galactosidase activity were calculated using the following formula: units of β-galactosidase activity = (1000 x OD_420_) / (V x t x OD_600_), where V = the volume of cells (ml); t = the incubation time (min); OD_600_ = optical density at the beginning of the experiment.

### Bimolecular fluorescence complementation (BiFC) assays

The PRC2 components (AT5G51230 (EMF2) and AT5G58230 (MSI1)), the candidate transcriptional regulators AT1G29300 (CHIQL6), AT2G45260 (CHIQ1), and AT1G53380 (CHIQL5), and a pulled-down transcription factor AT3G23690 (bHLH077) in pUC-SPV-NE^GW^ and pUC-SPV-CE^GW^ vectors (described in “Construction of plasmids”) were used for BiFC assays in Arabidopsis protoplasts as previously described [119, 120]. For each experiment, the Venus signal was compared only within the protoplast populations prepared and transformed at the same time. Images were taken with a confocal microscope with the same gain (Leica, LCS SL). Multiple images were taken for each biological replicate. The interaction frequency was calculated by counting the number of Venus positive nuclei among all protoplasts under an epifluorescence microscope (Olympus, MVX100). At least 250 protoplasts were counted for each sample in each experiment and three independent experiments were performed for each combination tested.

### Pull-down assays

Fully expanded 3rd, 4th, or 5th tobacco leaves from 5 to 6 week-old plants were co-infiltrated with the following combinations: GFP-tagged CHIQ1 plus FLAG-tagged CHIQL6; GFP-tagged CHIQ1 plus FLAG-tagged CHIQL5; GFP tagged CHIQ1 plus FLAG-tagged EMF2; and FLAG-tagged CHIQL6 plus GST-tagged EMF2. Three days after infiltration, the leaves were collected, frozen in liquid nitrogen, and kept at −80 °C.

For the pull-down assays, each tobacco leaf was ground using liquid nitrogen, and total protein content was extracted using a native buffer (100 mM sodium phosphate, pH 8.0 (Sigma-Aldrich), 150 mM sodium chloride (EMD), 5 mM EDTA (Sigma-Aldrich), 5 mM EGTA, 0.05% Triton X-100 (Sigma-Aldrich), and one tablet of cOmplete ULTRA protease inhibitor cocktail per 10 ml buffer (Roche)). Protein concentration of each extract was measured using Bradford assay (Bio-Rad). Ten micrograms of the anti-FLAG antibody (F3165, Sigma-Aldrich) were coupled to 50 μl of protein A/G magnetic beads (Thermo Scientific) following the manufacturer’s instructions.

Approximately 1 mg of total protein was incubated with the antibody-coupled beads for 1.5 h at 4 °C with gentle rotation. The beads were washed four times with the extraction buffer and the protein complexes were eluted with Laemmli buffer (Bio-Rad). The eluate was run in a 7.5% SDS-PAGE gel (Bio-Rad) and the IP was verified by Western blot using anti-FLAG HFR-coupled antibodies (Sigma-Aldrich), anti-GFP (Clontech), or anti-GST antibodies (donated by Dr. Z. Wang (Carnegie Institution for Science, USA)).

## Additional file

Additional file 1: 
**Tables S1**, **S3** and **S4** list the candidate transcriptional regulators predicted in Arabidopsis, fruit fly, and human, respectively. **Table S2** shows the number of families predicted from the genome by each criterion and the enrichment fold yield by each criteria towards the identification of regulators in Arabidopsis, fruit fly and human. **Table S5** lists the physical interactions between predicted regulators and proteins involved in transcription available in the BioGRID database [108] and determined in this study. **Table S6** shows the results of the segregation analysis of *chiq1–1* phenotype (dwarfism) in the F_2_ populations of chiq1–1 x Col-0 (wild type) crosses. **Table S7** shows the results of the linkage analysis of *chiq1–1* phenotype (dwarfism) and genotype in the F_2_ populations of *chiq1–1* x Col-0 (wild type) crosses. **Table S8** lists the proteins that co-immunoprecipitated (Co-IP/MS) with CHIQ1-GFP in vivo. **Table S9** lists the physical interactions among nine CHIQ proteins. **Figure S1** illustrates the pipeline workflow and the number of predictions in yeast, fruit fly and human. **Figure S2** shows the proportion of unknown genes in families with less than three or more than two members in Arabidopsis, yeast, fruit fly and human and the proportion of the predictions among the unknown families with more than two members. **Figure S3** shows the precision, recall and F1 score of TF predictions in Arabidopsis. **Figure S4** shows the maximum number of aspartic acid, glutamic acid, asparagine, glutamine, serine, proline and acidic amino acids in all proteins, TFs and the predicted regulators in Arabidopsis, fruit fly and human. **Figure S5** shows the GUS activity of the negative controls for the *in planta* transactivation assay. **Figure S6** shows the number of leaves at different ages and the age of bolting in wild type (Col-0), *chiq1–1* and B12 (complemented line). (DOCX 1780 kb)
