# Supplementary material for: Systematic discovery of novel eukaryotic transcriptional regulators using sequence homology independent prediction
Source: BMC Genomics. 2017 Jun 26;18:480. doi: 10.1186/s12864-017-3853-9 (PMC5485742; doi:10.1186/s12864-017-3853-9)
Supplement: Additional file 1: — Tables S1, S3 and S4 list the candidate transcriptional regulators predicted in Arabidopsis, fruit fly, and human, respectively. Table S2 shows the number of families predicted from the genome by each criterion and the enrichment fold yield by each criteria towards the identification of regulators in Arabidopsis, fruit fly and human. Table S5 lists the physical interactions between predicted regulators and proteins involved in transcription available in the BioGRID database [108] and determined in this study. Table S6 shows the results of the segregation analysis of chiq1–1 phenotype (dwarfism) in the F2 populations of chiq1–1 x Col-0 (wild type) crosses. Table S7 shows the results of the linkage analysis of chiq1–1 phenotype (dwarfism) and genotype in the F2 populations of chiq1–1 x Col-0 (wild type) crosses. Table S8 lists the proteins that co-immunoprecipitated (Co-IP/MS) with CHIQ1-GFP in vivo. Table S9 lists the physical interactions among nine CHIQ proteins. Figure S1 illustrates the pipeline workflow and the number of predictions in yeast, fruit fly and human. Figure S2 shows the proportion of unknown genes in families with less than three or more than two members in Arabidopsis, yeast, fruit fly and human and the proportion of the predictions among the unknown families with more than two members. Figure S3 shows the precision, recall and F1 score of TF predictions in Arabidopsis. Figure S4 shows the maximum number of aspartic acid, glutamic acid, asparagine, glutamine, serine, proline and acidic amino acids in all proteins, TFs and the predicted regulators in Arabidopsis, fruit fly and human. Figure S5 shows the GUS activity of the negative controls for the in planta transactivation assay. Figure S6 shows the number of leaves at different ages and the age of bolting in wild type (Col-0), chiq1–1 and B12 (complemented line). (DOCX 1780 kb) [file 12864_2017_3853_MOESM1_ESM.docx]

**Table S1.** List of putative transcriptional regulators predicted in *Arabidopsis thaliana.*

| **Gene ID** | **Cluster ID** | **DUF domain** | **Independent evidence supporting transcriptional regulator function** | **Reported phenotype of the null mutant, unless stated otherwise** | **Mutant lines analyzed in our study** |
| --- | --- | --- | --- | --- | --- |
| AT4G36100 | 35 | DUF641 |  |  |  |
| AT2G30380 | 35 | DUF641 |  |  |  |
| AT2G45260 | 35 | DUF641 |  |  | SALK_064001C |
| AT4G34080 | 35 | DUF641 |  |  |  |
| AT4G33320 | 35 | DUF641 |  |  |  |
| AT3G14870 | 35 | DUF641 |  |  |  |
| AT3G60680 | 35 | DUF641 |  |  |  |
| AT1G53380 | 35 | DUF641 |  |  |  |
| AT1G29300 | 35 | DUF641 |  | Defects in pollen tube attraction [[1](#_ENREF_1)] |  |
| AT5G58960 | 35 | DUF641 |  | Upward hypocotyl growth under red light [[2](#_ENREF_2)] |  |
| AT2G32130 | 35 | DUF641 |  |  |  |
| AT2G01260 | 42 | DUF789 |  | Affected in leaf phenotype [[3](#_ENREF_3)] |  |
| AT1G15030 | 42 | DUF789 |  |  |  |
| AT5G49220 | 42 | DUF789 |  |  |  |
| AT4G16100 | 42 | DUF789 |  |  |  |
| AT1G03610 | 42 | DUF789 |  |  |  |
| AT4G03420 | 42 | DUF789 |  |  |  |
| AT4G28150 | 42 | DUF789 |  |  |  |
| AT1G73210 | 42 | DUF789 |  |  |  |
| AT1G17830 | 42 | DUF789 |  |  |  |
| AT5G23380 | 42 | DUF789 |  |  |  |
| AT5G08360 | 42 | DUF789 |  |  |  |
| AT2G38820 | 51 | DUF506 |  |  |  |
| AT3G22970 | 51 | DUF506 |  |  |  |
| AT3G54550 | 51 | DUF506 |  |  |  |
| AT4G14620 | 51 | DUF506 |  |  | SALK_047914C |
| AT2G39650 | 51 | DUF506 |  |  |  |
| AT3G07350 | 51 | DUF506 |  |  |  |
| AT3G25240 | 51 | DUF506 |  |  |  |
| AT1G21475 | 51 | DUF506 |  |  |  |
| AT2G31160 | 52 | DUF640 | Belongs to the ALOG family [[4](#_ENREF_4)] | Overexpression of OBO1 causes an abnormal number and size of petals and petal-stamen fusions [[5](#_ENREF_5)] |  |
| AT3G23290 | 52 | DUF640 | Belongs to the ALOG family [[4](#_ENREF_4)] | Constitutive expression of LSH4 in the shoot apex causes inhibition of leaf growth in the vegetative phase, and formation of extra shoots or shoot organs within a flower in the reproductive phase [[6](#_ENREF_6)]  Mutant line is affected in heat and salt tolerance [[7](#_ENREF_7)] |  |
| AT1G07090 | 52 | DUF640 | Belongs to the ALOG family [[4](#_ENREF_4)] |  |  |
| AT5G28490 | 52 | DUF640 | Belongs to the ALOG family [[4](#_ENREF_4)] | Shorter hypocotyls and larger cotyledons under continuous light, and shorter cells in the hypocotyl. Shorter petioles in rosette leaves [[8](#_ENREF_8)] |  |
| AT3G04510 | 52 | DUF640 | Belongs to the ALOG family [[4](#_ENREF_4)] |  |  |
| AT5G58500 | 52 | DUF640 | Belongs to the ALOG family [[4](#_ENREF_4)] |  |  |
| AT2G42610 | 52 | DUF640 | Belongs to the ALOG family [[4](#_ENREF_4)] |  |  |
| AT1G78815 | 52 | DUF640 | Belongs to the ALOG family [[4](#_ENREF_4)] |  |  |
| AT4G18610 | 52 | DUF640 | Belongs to the ALOG family [[4](#_ENREF_4)] |  |  |
| AT1G16910 | 52 | DUF640 | Belongs to the ALOG family [[4](#_ENREF_4)] |  |  |
| AT2G32910 | 105 |  |  |  |  |
| AT5G61910 | 105 |  |  |  |  |
| AT2G35140 | 105 |  |  |  |  |
| AT3G11000 | 105 |  |  |  |  |
| AT5G42050 | 105 |  |  |  |  |
| AT5G01660 | 105 |  |  |  |  |
| AT3G27090 | 105 |  |  |  |  |
| AT4G37540 | 143 | DUF260 | LOB transcription factor family [[9](#_ENREF_9)] | Elevated anthocyanin levels in response to nitrogen [[10](#_ENREF_10)] |  |
| AT5G67420 | 143 | DUF260 | LOB transcription factor family [[9](#_ENREF_9)] | Elevated anthocyanin levels in response to nitrogen [[10](#_ENREF_10)] |  |
| AT3G49940 | 143 | DUF260 | LOB transcription factor family [[9](#_ENREF_9)] | Elevated anthocyanin levels in response to nitrogen [[10](#_ENREF_10)] |  |
| AT3G02550 | 143 | DUF260 | LOB transcription factor family [[9](#_ENREF_9)] |  |  |
| AT1G67100 | 143 | DUF260 | LOB transcription factor family [[9](#_ENREF_9)] |  |  |
| AT1G68510 | 143 | DUF260 | LOB transcription factor family [[9](#_ENREF_9)] |  |  |
| AT2G33780 | 164 |  | VQ motif containing protein family [[11](#_ENREF_11)][15] |  |  |
| AT1G28280 | 164 |  | VQ motif containing protein family [[11](#_ENREF_11)][15] |  |  |
| AT3G15300 | 164 |  | VQ motif containing protein family [[11](#_ENREF_11)][15] |  |  |
| AT5G53830 | 164 |  | VQ motif containing protein family [[11](#_ENREF_11)][15] |  |  |
| AT5G08480 | 164 |  | VQ motif containing protein family [[11](#_ENREF_11)][15] |  |  |
| AT1G80450 | 164 |  | VQ motif containing protein family [[11](#_ENREF_11)][15] |  |  |
| AT4G32342 | 175 |  |  |  |  |
| AT5G25360 | 175 |  |  |  |  |
| AT1G15350 | 175 |  |  |  |  |
| AT3G15770 | 175 |  |  |  |  |
| AT3G54880 | 175 |  |  |  |  |
| AT5G03440 | 175 |  |  |  |  |
| AT1G62390 | 207 |  |  | Chloroplast and non-green plastid clustering. Flowers slightly earlier than wild type [[12](#_ENREF_12)] |  |
| AT5G20360 | 207 |  |  |  |  |
| AT4G32070 | 207 |  |  |  |  |
| AT2G25290 | 207 |  |  |  |  |
| AT3G16760 | 207 |  |  |  |  |
| AT2G35230 | 261 |  | VQ motif containing protein family [[15](#_ENREF_15)] | Smaller seeds [[13](#_ENREF_13), [14](#_ENREF_14)] |  |
| AT1G32610 | 261 |  | VQ motif containing protein family [[15](#_ENREF_15)] |  |  |
| AT5G46780 | 261 |  | VQ motif containing protein family [[15](#_ENREF_15)] |  |  |
| AT1G78310 | 261 |  | VQ motif containing protein family [[15](#_ENREF_15)] |  |  |
| AT1G32585 | 261 |  | VQ motif containing protein family [[15](#_ENREF_15)] |  |  |
| AT4G10480 | 273 |  | Belongs to the NACA protein family [[16](#_ENREF_16)] |  |  |
| AT1G33040 | 273 |  | Belongs to the NACA protein family [[16](#_ENREF_16)] |  |  |
| AT3G49470 | 273 |  | Belongs to the NACA protein family [[16](#_ENREF_16)] |  |  |
| AT3G12390 | 273 |  | Belongs to the NACA protein family [[16](#_ENREF_16)] |  |  |
| AT5G13850 | 273 |  | Belongs to the NACA protein family [[16](#_ENREF_16)] |  |  |
| AT4G18150 | 279 | DUF1296 |  |  |  |
| AT1G29370 | 279 | DUF1296 |  |  |  |
| AT1G29350 | 279 | DUF1296 |  |  |  |
| AT5G46380 | 279 | DUF1296 |  |  |  |
| AT3G13990 | 279 | DUF1296 |  |  | SALK_000579C |
| AT2G44440 | 305 |  | Belongs to the EMSY-LIKE protein family [[17-22](#_ENREF_17)] | Affected in defense responses (race-specific immunity and/or basal) [[23](#_ENREF_23)] |  |
| AT5G13020 | 305 |  | Belongs to the EMSY-LIKE protein family [[17-22](#_ENREF_17)] | Affected in defense responses (race-specific immunity and/or basal) [[23](#_ENREF_23)] |  |
| AT3G12140 | 305 |  | Belongs to the EMSY-LIKE protein family [[17-22](#_ENREF_17)] | Affected in defense responses (race-specific immunity and/or basal) [[23](#_ENREF_23)] |  |
| AT5G06780 | 305 |  | Belongs to the EMSY-LIKE protein family [[17-22](#_ENREF_17)] | Affected in defense responses (race-specific immunity and/or basal) [[23](#_ENREF_23)] |  |
| AT3G57960 | 305 |  |  |  |  |
| AT3G57970 | 305 |  |  |  |  |
| AT4G04630 | 356 | DUF584 |  |  |  |
| AT4G21970 | 356 | DUF584 |  |  |  |
| AT3G15040 | 356 | DUF584 |  |  |  |
| AT5G04820 | 392 |  | Belongs to the OVATE transcription factor family [[24](#_ENREF_24)] | Overexpressor plants had blunt-ended siliques [[24](#_ENREF_24)] |  |
| AT2G36050 | 392 |  | Belongs to the OVATE transcription factor family [[24](#_ENREF_24)] | Overexpressor plants had blunt-ended siliques [[24](#_ENREF_24)] |  |
| AT3G52540 | 392 |  | Belongs to the OVATE transcription factor family [[24](#_ENREF_24)] | Overexpressor plants had blunt-ended siliques [[24](#_ENREF_24)] |  |
| AT3G52550 | 392 |  | Belongs to the OVATE transcription factor family [[24](#_ENREF_24)] |  |  |
| AT4G18692 | 436 |  |  |  |  |
| AT3G53342 | 436 |  |  |  |  |
| AT1G55710 | 436 |  |  |  |  |
| AT3G53630 | 436 |  |  | Affected in oxidative and osmotic tolerance [[7](#_ENREF_7)] |  |
| AT1G70420 | 479 | DUF1645 |  |  |  |
| AT1G23710 | 479 | DUF1645 |  |  | SALK_043683C |
| AT3G27880 | 479 | DUF1645 |  |  |  |
| AT5G62770 | 479 | DUF1645 |  |  |  |
| AT2G40435 | 505 |  |  | Affected in osmotic tolerance [[7](#_ENREF_7)] |  |
| AT3G56220 | 505 |  |  |  |  |
| AT1G29270 | 505 |  |  |  |  |
| AT2G16575 | 533 |  |  |  |  |
| AT1G17780 | 533 |  |  |  |  |
| AT1G73130 | 533 |  |  |  |  |
| AT2G41730 | 544 |  |  |  |  |
| AT5G24640 | 544 |  |  |  |  |
| AT5G40690 | 544 |  |  |  |  |
| AT2G34170 | 548 | DUF688 |  |  |  |
| AT1G29240 | 548 | DUF688 |  |  |  |
| AT2G30990 | 548 | DUF688 |  |  |  |
| AT2G01990 | 576 |  |  |  |  |
| AT1G14630 | 576 |  |  | Affected in osmotic tolerance [[7](#_ENREF_7)] |  |
| AT5G48720 | 576 |  |  | Sterile due to extensive chromosome fragmentation [[25](#_ENREF_25)]  50% defective seeds [[1](#_ENREF_1)] |  |
| AT4G21930 | 599 | DUF584 | Activation activity *in planta* (this study) |  |  |
| AT1G11700 | 599 | DUF584 |  |  |  |
| AT1G61930 | 599 | DUF584 |  |  |  |
| AT4G26950 | 599 | DUF584 |  |  |  |
| AT2G37570 | 606 |  |  |  |  |
| AT5G02480 | 606 |  |  |  |  |
| AT3G12570 | 606 |  |  |  |  |
| AT2G19390 | 655 |  |  |  |  |
| AT4G29790 | 655 |  |  |  |  |
| AT5G22450 | 655 |  |  |  |  |
| AT4G28300 | 699 | DUF1421 |  |  | SALK_048257C |
| AT5G14540 | 699 | DUF1421 |  |  | SALK_145362C  SALK_103773C |
| AT3G01560 | 699 | DUF1421 |  |  |  |
| AT1G64140 | 707 |  |  |  |  |
| AT5G09670 | 707 |  |  |  |  |
| AT5G64550 | 707 |  |  |  |  |
| AT4G37440 | 717 |  |  |  |  |
| AT3G59670 | 717 |  | Activation activity *in planta* (this study) |  |  |
| AT3G50040 | 717 |  |  |  |  |
| AT4G21460 | 729 |  |  |  |  |
| AT3G18240 | 729 |  |  |  |  |
| AT1G73770 | 729 |  |  |  |  |
| AT4G24590 | 731 |  |  | Affected in cold, osmotic, salt and ABA tolerance [[7](#_ENREF_7)] |  |
| AT5G49710 | 731 |  |  |  |  |
| AT1G44770 | 731 |  |  |  |  |
| AT4G14840 | 743 |  |  |  |  |
| AT3G22520 | 743 |  |  |  |  |
| AT1G05410 | 743 | DUF1423 |  |  | SALK_005912CSALK_061502C |
| AT4G08910 | 760 |  |  |  |  |
| AT1G78170 | 760 |  |  |  |  |
| AT1G22250 | 760 |  |  | Affected in heat, oxidative, and salt tolerance [[7](#_ENREF_7)] |  |
| AT1G18620 | 831 |  |  | Affected in cold tolerance [[7](#_ENREF_7)] |  |
| AT1G74160 | 831 |  |  |  |  |
| AT3G63430 | 831 |  |  |  |  |
| AT1G67250 | 835 |  |  |  |  |
| AT5G38650 | 835 |  | Activation activity *in planta* (this study) |  |  |
| AT1G62920 | 835 |  |  |  |  |
| AT1G80040 | 845 |  |  |  |  |
| AT5G32440 | 845 |  | Activation activity *in planta* (this study) |  |  |
| AT5G02510 | 845 |  |  | Decreased rosette relative size, leaf relative size, and petiole relative size. Pale green leaves [[3](#_ENREF_3)] |  |
| AT3G49140 | 850 |  |  |  |  |
| AT5G24060 | 850 |  |  |  |  |
| AT3G59300 | 850 |  |  |  |  |
| AT3G26990 | 871 | DUF618 | Activation activity *in planta* (this study) |  |  |
| AT5G65180 | 871 | DUF618 |  |  |  |
| AT5G10060 | 871 | DUF618 |  |  |  |
| AT1G13970 | 99999 | DUF1336 |  |  |  |
| AT3G29180 | 99999 | DUF1336 |  |  |  |
| AT5G39430 | 99999 | DUF1336 |  |  |  |
| AT1G59650 | 99999 | DUF1336 |  |  |  |
| AT1G10410 | 99999 | DUF1336 |  | Affected in oxidative and ABA tolerance [[7](#_ENREF_7)] |  |
| AT2G28140 | A1 | DUF1635 |  |  |  |
| AT5G22930 | A1 | DUF1635 |  |  |  |
| AT3G44940 | A1 | DUF1635 |  |  |  |
| AT2G28690 | A1 | DUF1635 |  |  |  |
| AT5G59760 | A1 | DUF1635 |  |  |  |
| AT5G09940 | A1 | DUF1635 |  |  |  |
| AT5G16780 | A2 |  |  | Dwarf plants that display an aberrant spurred leaf venation pattern and fail to flower [[26](#_ENREF_26)] |  |
| AT3G63095 | A2 |  |  |  |  |
| AT3G14700 | A2 |  |  |  |  |
| AT2G36640 | A3 |  |  | Affected in heat tolerance [[7](#_ENREF_7)] |  |
| AT2G42560 | A3 |  |  |  |  |
| AT3G53040 | A3 |  |  |  |  |
| AT3G12890 | A4 |  | Transcriptional activity *in planta.*  CCT motif family protein [[27](#_ENREF_27)]  Annotated as TR family in [[28](#_ENREF_28)] |  |  |
| AT2G33350 | A4 |  | CCT motif family protein [[27](#_ENREF_27)]  Annotated as TR family in [[28](#_ENREF_28)] |  |  |
| AT1G04500 | A4 |  | CCT motif family protein [[27](#_ENREF_27)]  Annotated as TR family in [[28](#_ENREF_28)] |  |  |
| AT5G41380 | A4 |  | CCT motif family protein [[27](#_ENREF_27)]  Annotated as TR family in [[28](#_ENREF_28)] |  |  |
| AT1G63820 | A4 |  | CCT motif family protein [[27](#_ENREF_27)]  Annotated as TR family in [[28](#_ENREF_28)] |  |  |
| AT5G59990 | A4 |  | CCT motif family protein [[27](#_ENREF_27)]  Annotated as TR family in [[28](#_ENREF_28)] |  |  |
| AT4G25210 | A5 | DUF573 | Belongs to the GeBP family [[29](#_ENREF_29), [30](#_ENREF_30)] |  |  |
| AT4G01260 | A5 | DUF573 | Belongs to the GeBP family [[29](#_ENREF_29), [30](#_ENREF_30)] |  |  |
| AT5G23180 | A5 |  |  |  |  |
| AT4G00130 | A5 | DUF573 |  |  |  |
| AT1G53935 | A5 |  |  |  |  |

Notes:

DUF640 ([PF04852](http://pfam.sanger.ac.uk/family/PF04852)) is annotated as ALOG domain (IPR006936) in the Interpro database.

DUF260 ([PF03195](http://pfam.sanger.ac.uk/family/PF03195)) is annotated as Lateral organ boundaries (LOB) (IPR004883) in the Interpro database.

**Table S2.** Number of families predicted from the genome by each criterion and enrichment fold yield by each criteria towards the identification of regulators in Arabidopsis, fruit fly and human

|  | Arabidopsis | | Fruit fly | | Human | |
| --- | --- | --- | --- | --- | --- | --- |
|  | Number of families | Fold enrichment | Number of families | Fold enrichment | Number of families | Fold enrichment |
| Genome | 8438 | - | 6698 | - | 7366 | - |
| Autoactivation-tested | 3970 | - | 3978 | - | 6118 | - |
| **Features in the pipeline:** | | | | | | |
| Unknown molecular function | 4604 | 1.8 | 3489 | 1.9 | 2318 | 3.2 |
| More than 3 members | 2734 | 3.1 | 1028 | 6.5 | 1911 | 3.9 |
| High percentage of disordered amino acids | 5059 | 1.7 | 4084 | 1.6 | 4546 | 1.6 |
| Nuclear localization | 2604 | 3.2 | 2174 | 3.1 | 2518 | 2.9 |
| Yeast autoactivation | 813 | 4.9 | 596 | 6.7 | 682 | 9.0 |
| Candidates | 43 | 92.3 | 7 | 568.3 | 9 | 679.8 |

**
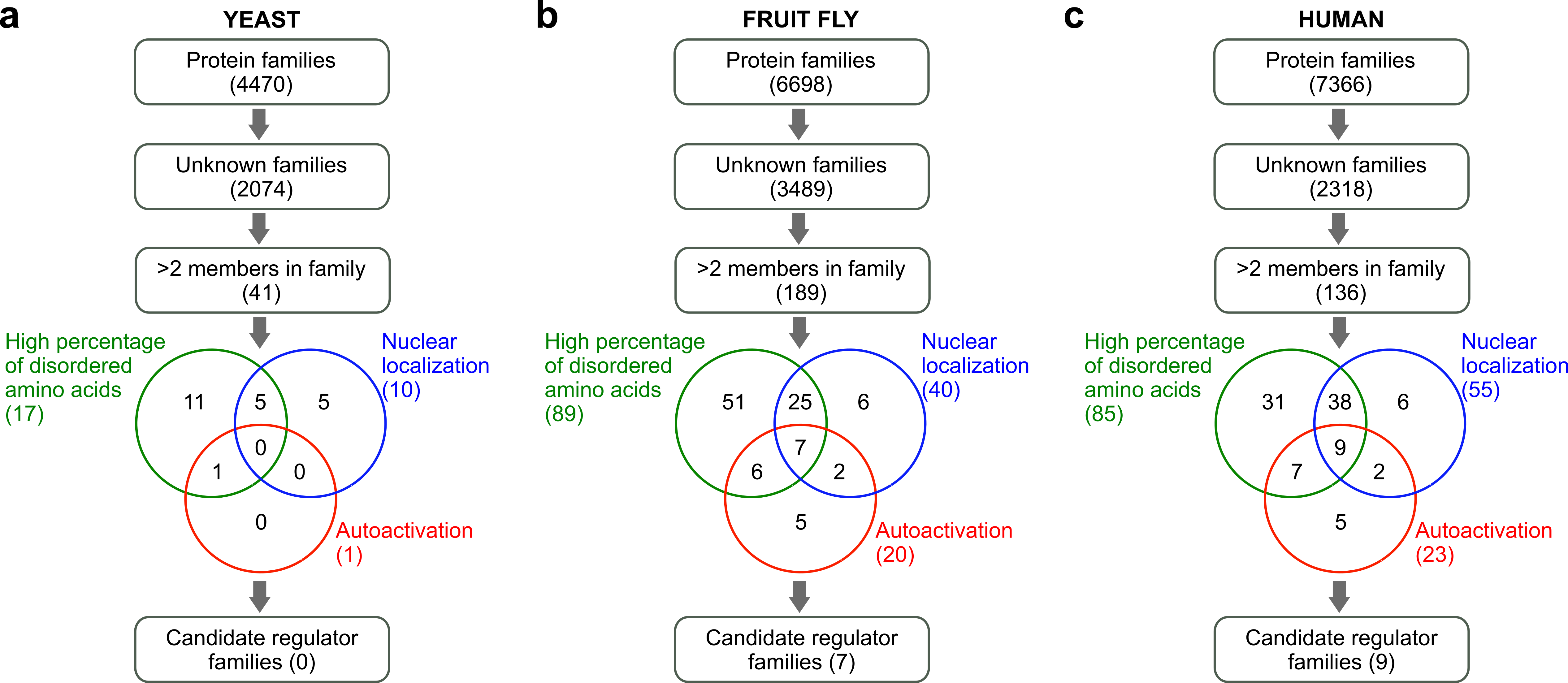
**

**Figure S1.** Pipeline workflow to identify novel transcriptional regulator families in yeast, fruit fly and human

Families with uncharacterized proteins in yeast (**a**), fruit fly (**b**), and human (**c**) were filtered by size. The numbers in the Venn diagram represents the number of families with most members being nuclear localized (blue), high percentage of disordered residues (green), and the ability of at least one member to activate transcription of a reporter gene in yeast (autoactivation, red). Families in the intersection of all three Venn diagrams were considered candidate regulator families.

**Table S3.** List of putative transcriptional regulators predicted in *Drosophila melanogaster.*

| **Gene ID** | **Cluster ID** | **Independent evidence supporting transcriptional regulator function** | **Mutant phenotype** |
| --- | --- | --- | --- |
| FBgn0030566 | 1 | Belongs to the NACA protein family [[16](#_ENREF_16)] |  |
| FBgn0030538 | 1 | Belongs to the NACA protein family [[16](#_ENREF_16)] |  |
| FBgn0030563 | 1 | Belongs to the NACA protein family [[16](#_ENREF_16)] |  |
| FBgn0052598 | 1 | Belongs to the NACA protein family [[16](#_ENREF_16)] |  |
| FBgn0052601 | 1 | Belongs to the NACA protein family [[16](#_ENREF_16)] |  |
| FBgn0031514 | 2 |  |  |
| FBgn0259150 | 2 |  |  |
| FBgn0259994 | 2 |  |  |
| FBgn0031545 | 3 |  |  |
| FBgn0031546 | 3 |  | Neurogenesis [[31](#_ENREF_31)] |
| FBgn0035097 | 3 |  |  |
| FBgn0031722 | 4 |  | Lateral inhibition [[32](#_ENREF_32)] |
| FBgn0031723 | 4 |  |  |
| FBgn0031724 | 4 |  |  |
| FBgn0032293 | 5 | Participates in histone H3-K4 methylation [[33](#_ENREF_33)] | Lateral inhibition [[32](#_ENREF_32)] |
| FBgn0035491 | 5 |  | Sperm motility [[34](#_ENREF_34)] |
| FBgn0053060 | 5 |  |  |
| FBgn0032636 | 6 |  |  |
| FBgn0032637 | 6 |  |  |
| FBgn0029705 | 6 |  |  |
| FBgn0036152 | 7 | Contains MADF sequence-specific DNA binding domain [[35](#_ENREF_35)] | Imaginal disc-derived wing morphogenesis [[36](#_ENREF_36)]  Inter-male aggressive behavior [[37](#_ENREF_37)]  Regulation of cell cycle [[38](#_ENREF_38)] |
| FBgn0039733 | 7 | Contains MADF sequence-specific DNA binding domain [[35](#_ENREF_35)] |  |
| FBgn0034945 | 7 | Contains MADF sequence-specific DNA binding domain [[35](#_ENREF_35)] |  |

**Table S4.** List of putative transcriptional regulators predicted in *Homo sapiens.*

| **Gene ID** | **UnirProtKB ID** | **Cluster ID** | **Independent evidence supporting transcriptional regulator function** | **Mutant phenotype/ Association to diseases** | **Comment** |
| --- | --- | --- | --- | --- | --- |
| NBP6L_HUMAN | A8MWS5 | 1 |  |  | On September 3, 2014 this entry was deleted. |
| NBPF5_HUMAN | Q86XG9 | 1 |  |  |  |
| NBPF7_HUMAN | P0C2Y1 | 1 |  |  |  |
| NBPF4_HUMAN | Q96M43 | 1 |  |  |  |
| NBPF6_HUMAN | Q5VWK0 | 1 |  |  |  |
| NBPFK_HUMAN | Q3BBV1 | 1 |  |  |  |
| NBPF1_HUMAN | Q3BBV0 | 1 |  | This gene is interrupted by constitutional translocation in a patient with neuroblastoma [[39](#_ENREF_39)] |  |
| NBPFL_HUMAN | A6NDD8 | 1 |  |  | On September 3, 2014 this entry was deleted. |
| NBPF9_HUMAN | Q3BBW0 | 1 |  |  |  |
| NBPFN_HUMAN | Q86T75 | 1 |  |  | Merged with NBPFB_HUMAN |
| NBPFA_HUMAN | Q6P3W6 | 1 |  |  |  |
| NBPFB_HUMAN | Q86T75 | 1 |  |  |  |
| NBPF8_HUMAN | Q3BBV2 | 1 |  |  |  |
| NBPFF_HUMAN | Q8N660 | 1 |  |  |  |
| NBPFG_HUMAN | Q5SXJ2 | 1 |  |  | Merged with Q8N660 |
| NBPFE_HUMAN | Q5TI25 | 1 |  |  |  |
| NBPFP_HUMAN | B4DH59 | 1 |  |  |  |
| NBPF3_HUMAN | Q9H094 | 1 |  |  |  |
| NBPFC_HUMAN | Q5TAG4 | 1 |  |  |  |
| FA83A_HUMAN | Q86UY5 | 2 |  | Novel tumor-specific gene that is highly expressed in human lung adenocarcinoma [[40](#_ENREF_40)] |  |
| FA83C_HUMAN | Q9BQN1 | 2 |  |  |  |
| FA83H_HUMAN | Q6ZRV2 | 2 |  | Amelogenesis imperfecta [[41](#_ENREF_41)] |  |
| FA83G_HUMAN | A6ND36 | 2 |  | Participates in the bone morphogenetic protein (BMP) signaling[[42](#_ENREF_42)], whose malfunction associates with several human diseases, including bone and developmental defects as well as cancer [[43](#_ENREF_43)] |  |
| FA83B_HUMAN | Q5T0W9 | 2 |  |  |  |
| FA83F_HUMAN | Q8NEG4 | 2 |  |  |  |
| FA83D_HUMAN | Q9H4H8 | 2 |  |  |  |
| FA83E_HUMAN | Q2M2I3 | 2 |  |  |  |
| C144C_HUMAN | Q8IYA2 | 3 |  |  |  |
| C144A_HUMAN | A2RUR9 | 3 |  |  |  |
| C144B_HUMAN | Q3MJ40 | 3 |  |  |  |
| C144L_HUMAN | Q6NUI1 | 3 |  |  |  |
| F122A_HUMAN | Q96E09 | 4 |  |  |  |
| F122B_HUMAN | Q7Z309 | 4 |  |  |  |
| F222C_HUMAN | Q6P4D5 | 4 |  |  |  |
| F134A_HUMAN | Q8NC44 | 5 |  |  |  |
| F134C_HUMAN | Q86VR2 | 5 |  |  |  |
| F134B_HUMAN | Q9H6L5 | 5 |  | Severe sensory and autonomic neuropathy [[44](#_ENREF_44)] | Co-localizes with Golgi-matrix proteins [[45](#_ENREF_45)]. This might be a false positive. |
| F153A_HUMAN | Q9UHL3 | 6 |  |  |  |
| F153B_HUMAN | P0C7A2 | 6 |  |  |  |
| F153C_HUMAN | Q494X1 | 6 |  |  |  |
| FA53A_HUMAN | Q6NSI3 | 7 |  |  |  |
| FA53B_HUMAN | Q14153 | 7 |  |  |  |
| FA53C_HUMAN | Q9NYF3 | 7 |  | Associated to myeloid diseases [[46](#_ENREF_46)] |  |
| LCE1C_HUMAN | Q5T751 | 8 |  |  | Structural component of mature cornified envelopes in epithelial tissues [[47](#_ENREF_47), [48](#_ENREF_48)]. This might be a false positive. |
| LCE1B_HUMAN | Q5T7P3 | 8 |  |  | Structural component of mature cornified envelopes in epithelial tissues [[47](#_ENREF_47), [48](#_ENREF_48)]. This might be a false positive. |
| LCE1E_HUMAN | Q5T753 | 8 |  |  | Structural component of mature cornified envelopes in epithelial tissues [[47](#_ENREF_47), [48](#_ENREF_48)]. This might be a false positive. |
| TMC1_HUMAN | Q8TDI8 | 9 |  | Deafness [[49](#_ENREF_49)] | Component of the mechanosensitive ion channel [[50](#_ENREF_50)]. This is a false positive. |
| TMC2_HUMAN | Q8TDI7 | 9 |  |  | Component of the mechanosensitive ion channel [[50](#_ENREF_50)]. This is a false positive. |
| TMC3_HUMAN | Q7Z5M5 | 9 |  |  |  |


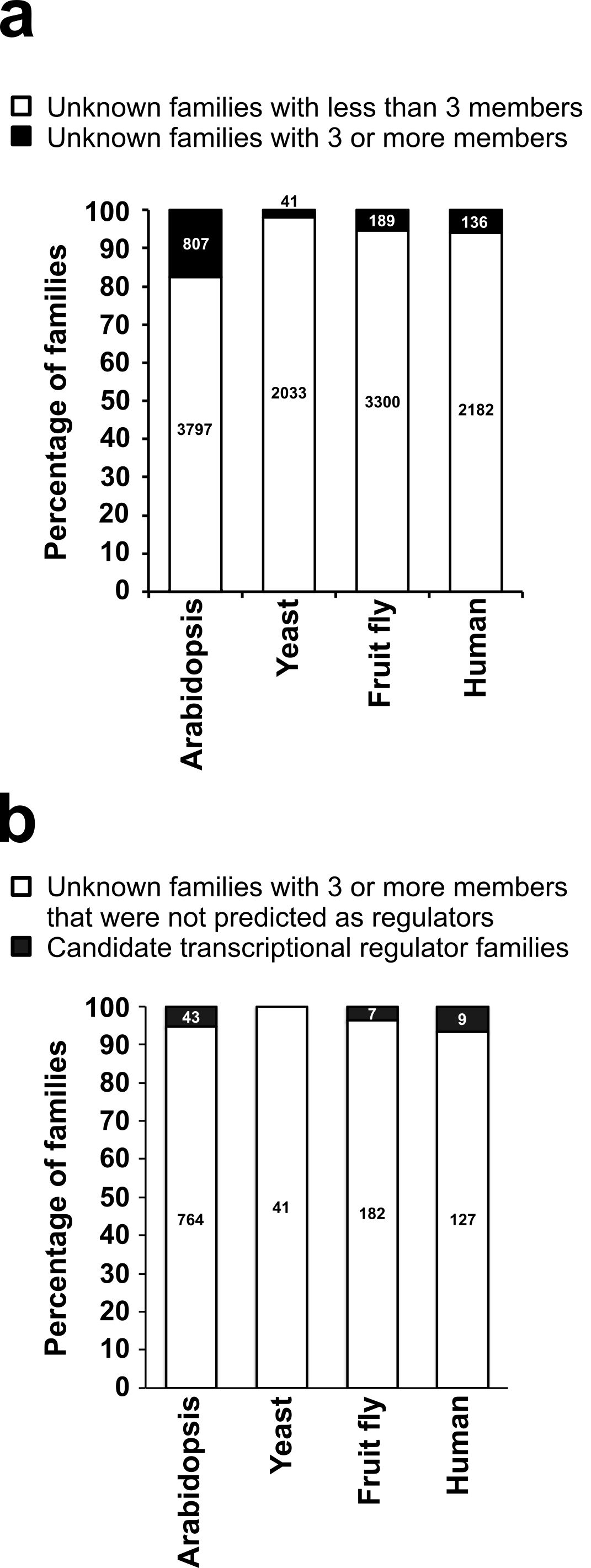


**Figure S2.**

**a**, Percentage of uncharacterized families with less than three (white) and more than two (black) members in Arabidopsis, yeast, fruit fly and human.

**b**, Percentage of candidate transcriptional regulators (black) that were predicted from the set of uncharacterized families with more than two members in Arabidopsis, yeast, fruit fly and human.

**
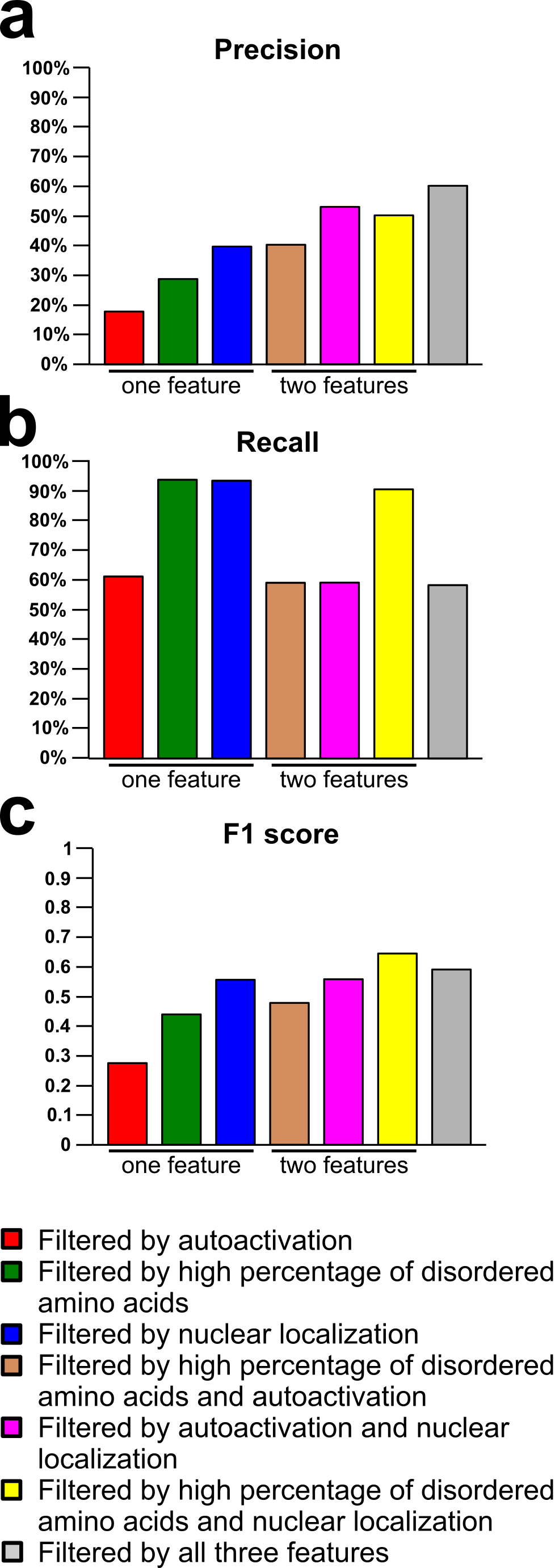
**

**Figure S3.** Precision, recall and F1 score of the predictions in Arabidopsis

Precision (**a**), recall (**b**), and the F1 score (**c**) of each feature individually (red, green, and blue bars) and all feature combinations (brown, magenta, yellow and gray bars) were calculated based on the number of TFs and TRs that were identified by the pipeline out of all predictions with GO annotations in Arabidopsis.

**
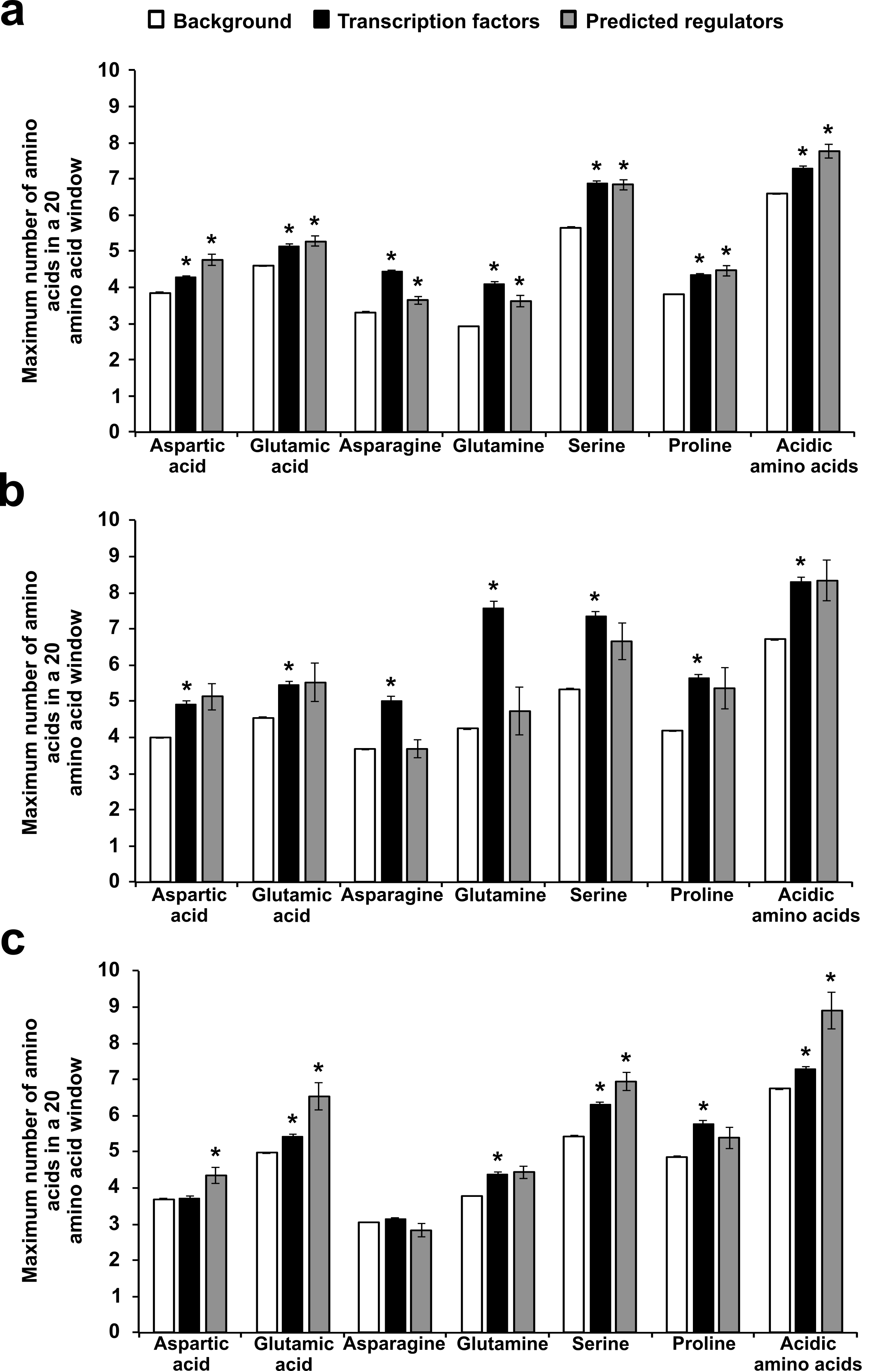
**

**Figure S4.** *In silico* analysis of the predictions.

**a-c,** The maximum number of aspartic acid, glutamic acid, asparagine, glutamine, serine, proline, and acidic amino acids in a 20 amino acid window in all proteins (white), TFs (black) and the predicted regulators (gray) in Arabidopsis (**a**), fruit fly (**b**) and human (**c**). * =: Bonferroni-corrected p-value < 0.005 when comparing TFs or the predicted regulators against all proteins in the genome (t-test). Error bars indicate standard error.

**Table S5.** Physical interactions between predicted regulators and proteins involved in transcription

| **Cluster** | **Gene ID** | **Interactor** | **Reference/s** |
| --- | --- | --- | --- |
| 52 | AT2G31160 | ARR-B transcription factors (ARR7, ARR14) | [[51](#_ENREF_51)] |
| 52 | AT5G28490 | ARR-B transcription factors (ARR6) | [[51](#_ENREF_51)] |
| 105 | AT5G42050 | Zinc finger protein, implicated in transcriptional regulation (CSP3) | [[52](#_ENREF_52)] |
| 143 | AT5G67420 | Transcriptional co-repressors (TPL, TPR1, TPR2, TPR3) | [[53](#_ENREF_53)] |
| 143 | AT3G02550 | Transcriptional co-repressors (TPL, TPR1, TPR2, TPR3) | [[53](#_ENREF_53)] |
| 164 | AT5G53830 | WRKY transcription factors (WRKY33, WRKY25) | [[15](#_ENREF_15)] |
| 164 | AT5G08480 | WRKY transcription factors (WRKY33, WRKY51) | [[15](#_ENREF_15)] |
| 164 | AT3G15300 | WRKY transcription factors (WRKY33, WRKY25) | [[15](#_ENREF_15)] |
| 164 | AT1G80450 | WRKY transcription factors (WRKY33, WRKY25) | [[15](#_ENREF_15)] |
| 164 | AT2G33780 | WRKY transcription factors (WRKY33, WRKY25) | [[15](#_ENREF_15)] |
| 164 | AT1G28280 | WRKY transcription factors (WRKY33, WRKY25) | [[15](#_ENREF_15)] |
| 261 | AT5G46780 | WRKY transcription factor (WRKY20) | [[54](#_ENREF_54)] |
| 261 | AT1G78310 | WRKY transcription factors (WRKY8, WRKY20) | [[54](#_ENREF_54), [55](#_ENREF_55)] |
| 261 | AT1G32585 | WRKY transcription factors (WRKY33, WRKY25, WRKY51) | [[15](#_ENREF_15)] |
| 273 | AT5G13850 | Homeobox transcription factor (WOX5) | [[56](#_ENREF_56)] |
| 279 | AT3G13990 | AP2-like ethylene-responsive transcription factor (TOE2) | [[54](#_ENREF_54)] |
| 305 | AT5G06780 | Homeobox transcription factor (SHH2) and B-ZIP transcription factor (TGA2) | [[54](#_ENREF_54)] |
| 392 | AT2G36050 | Homeobox transcription factors (BLH1, BLH3) | [[57](#_ENREF_57)] |
| 479 | AT5G62770 | WRKY transcription factor (WRKY60) | [[54](#_ENREF_54)] |
| 544 | AT2G41730 | MYB transcription factor and TCP transcription factors (TCP14, TCP15) | [[54](#_ENREF_54)] |
| A3 | AT2G42560 | TCP transcription factor (TCP14) | [[54](#_ENREF_54)] |
| 731 | AT5G49710 | ARR-B transcription factor (ARR14) | [[51](#_ENREF_51)] |
| 207 | AT4G32070 | Histone acetyltransferase GCN5 | [[58](#_ENREF_58)] |
| 143 | AT4G37540 | ATP-dependent helicase BRAHMA | [[59](#_ENREF_59)] |
| 143 | AT3G49940 | SWI/SNF complex subunit SWI3C | [[59](#_ENREF_59)] |
| 143 | AT1G67100 | Chromatin structure-remodeling complex protein SPLAYED | [[59](#_ENREF_59)] |
| 871 | AT5G10060 | Mediator subunit (MED31) | [[54](#_ENREF_54)] |
| A4 | AT4G25210 | A DEK domain-containing chromatin associated protein | [[60](#_ENREF_60)] |
| 35 | AT1G29300 | Polycomb repressive complex 2, subunit EMF2 | This study |
| 35 | AT2G32130 | Polycomb repressive complex 2, subunit CLF | This study |
| 35 | AT5G58960 | Polycomb repressive complex 2, subunits CLF and SWN | This study |
| 3 | FBgn0031545 | zf-C2H2 transcription factor | [[61](#_ENREF_61)] |
| 3 | FBgn0031546 | p53 and two zf-C2H2 transcription factors | [[62](#_ENREF_62)] |
| 3 | FBgn0035097 | Brahma associated protein 60kD, three zf-C2H2 transcription factors, TBP coactivator and a protein that [contributes to histone acetyltransferase activity](http://flybase.org/cgi-bin/cvreport.html?id=GO:0004402) | [[61](#_ENREF_61)] |
| 4 | FBgn0031722 | Six zf-C2H2 transcription factors | [[61](#_ENREF_61)] |
| 4 | FBgn0032293 | Set1/Ash2 histone methyltransferase complex subunit ASH2 and zf-C2H2 transcription factor | [[63](#_ENREF_63)] |
| 5 | FBgn0035491 | zf-C2H2 transcription factor | [[61](#_ENREF_61)] |
| 6 | FBgn0032637 | bHLH, bZIP, zf-BED, and zf-C2H2 transcription factors | [[61](#_ENREF_61)] |
| 1 | NBPFB_HUMAN | Transcriptional repressor (CBY1) | [[64](#_ENREF_64)] |
| 1 | NBPFF_HUMAN | Histone demethylase 1A (KDM1A) | [[65](#_ENREF_65)] |
| 1 | NBPF3_HUMAN | Transcriptional repressor (EWRS1) | [[66](#_ENREF_66)] |
| 2 | FA83H_HUMAN | bHLH and E2F transcription factors | [[67](#_ENREF_67), [68](#_ENREF_68)] |
| 2 | FA83G_HUMAN | Two MH1 transcription factor | [[69](#_ENREF_69)] |
| 2 | FA83D_HUMAN | bHLH and bZIP transcription factor | [[67](#_ENREF_67)] |
| 8 | LCE1B_HUMAN | Three Homeobox transcription factor | [[70](#_ENREF_70)] |


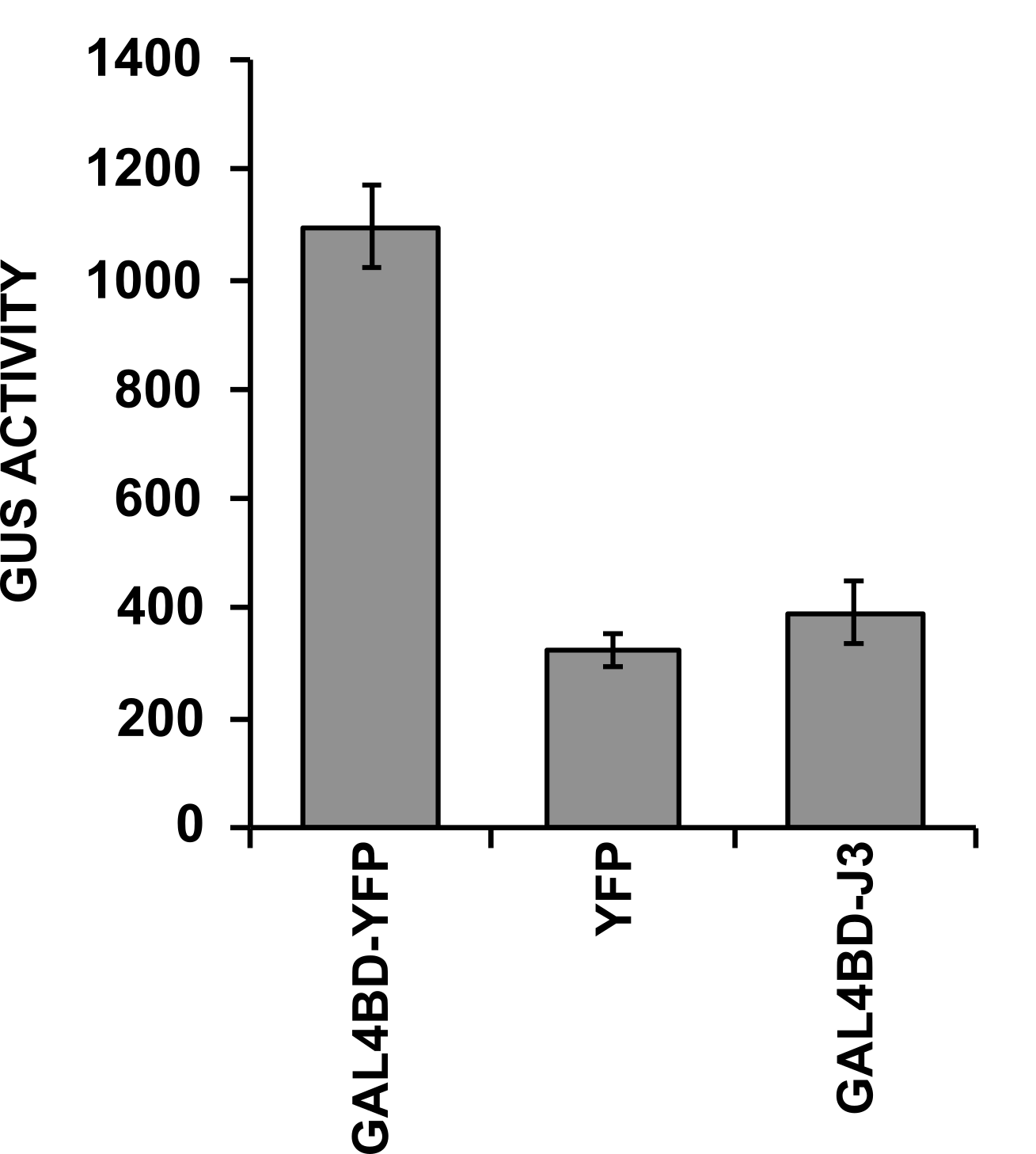


**Figure S5.** GUS activity of negative controls.

Average transactivation activity calculated as the GUS activity (nmol of 4MU/minute/mg total protein). Error bars represent standard error from at least 2 independent experiments.

**Table S6.** Segregation analysis of *chiq1-1* phenotype (dwarfism) in the F2 populations of at least three independent *chiq1-1* x Col-0 (wild type) crosses

| Phenotype | Short | Wild type |
| --- | --- | --- |
| Expected number of progeny | 31 | 94 |
| Observed number of progeny | 27 | 98 |

n = 125 plants; p-value: 0.65 (Fisher’s exact test)

**Table S7.** Linkage analysis of *chiq1-1* phenotype (dwarfism) and genotype in the F2 populations of at least three independent *chiq1-1* x Col-0 (wild type) crosses

| Genotype | *CHIQ1-1; CHIQ1-1* | *CHIQ1-1; chiq1-1* | *chiq1-1; chiq1-1* |
| --- | --- | --- | --- |
| Expected number of progeny | 15 | 29 | 15 |
| Observed number of progeny | 16 | 26 | 17 |
| Phenotype | 100% dwarf | 100% wild type | 100% wild type |

n = 59 plants; p-value: 0.82 (Fisher’s exact test)

**
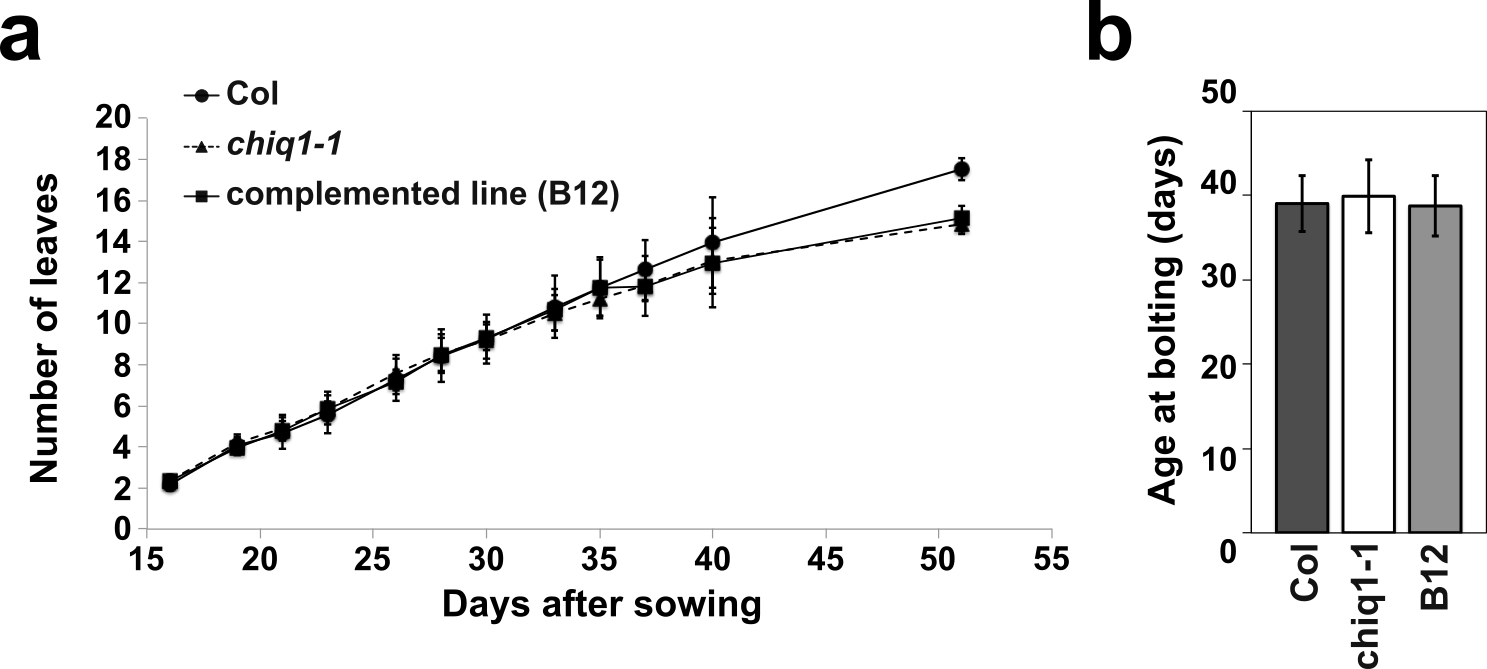
**

**Figure S6.** Developmental timing of *chiq1-1* plants

**a**, Number of leaves with a visible petiole from day 16 after sowing to day 39-40 in wild type (circles), *chiq1-1* (triangles and dashed line), and *chiq1-1* complemented with *CHIQ1* (B12, squares) plants grown in soil. (n = 9-12 per genotype per experiment from 8 independent experiments) **b**, Bolting time was determined as the number of days that passed between sowing and when the primary inflorescence was at least 1 cm tall for wild type (black), *chiq1-1* (white), and *chiq1-1* complemented with *CHIQ1* (B12, gray) plants. (n = 9-12 per genotype per experiment from 8 independent experiments). Error bars represent standard error from 8 independent experiments.

**Table S8.** Proteins that co-immunoprecipitated (Co-IP/MS) with CHIQ1-GFP *in vivo.* Transcription-associated proteins are highlighted in green and CHIQ1 family members are highlighted in yellow. The proteins are rank ordered based on abundance in the co-immunoprecipitated sample from the most (top) to the least (bottom).

| **TAIR ID** | **Locus description** | **CoIP/MS** | **Y2H** | **Pull downs** | **BiFC** |
| --- | --- | --- | --- | --- | --- |
| AT2G45260 | Plant protein of unknown function (DUF641), CHIQ1 | ✔ |  |  |  |
| AT3G16460 | JACALIN-RELATED LECTIN 34 (JAL34) | ✔ |  |  |  |
| AT5G02500 | HEAT SHOCK COGNATE PROTEIN 70-1 (HSC70-1) | ✔ |  |  |  |
| AT4G36520 | Chaperone DnaJ-domain superfamily protein | ✔ |  |  |  |
| AT3G18780 | ACTIN 2 (ACT2) | ✔ |  |  |  |
| AT3G04840 | Ribosomal protein S3Ae | ✔ |  |  |  |
| AT4G34670 | Ribosomal protein S3Ae | ✔ |  |  |  |
| AT1G33120 | Ribosomal protein L6 family | ✔ |  |  |  |
| AT4G10450 | Ribosomal protein L6 family | ✔ |  |  |  |
| AT2G36880 | METHIONINE ADENOSYLTRANSFERASE 3 (MAT3) | ✔ |  |  |  |
| AT4G01850 | S-ADENOSYLMETHIONINE SYNTHETASE 2 (SAM-2) | ✔ |  |  |  |
| AT5G56030 | HEAT SHOCK PROTEIN 81-2 (HSP81-2) | ✔ |  |  |  |
| AT1G75310 | AUXILIN-LIKE 1 (AUL1) | ✔ |  |  |  |
| AT1G53380 | Plant protein of unknown function (DUF641) | ✔ | ✔ | ✔ | ✔ |
| AT1G59610 | DYNAMIN-LIKE 3 (DL3) | ✔ |  |  |  |
| AT1G10290 | DYNAMIN-LIKE PROTEIN 6 (ADL6) | ✔ |  |  |  |
| AT3G10380 | SUBUNIT OF EXOCYST COMPLEX 8 (SEC8) | ✔ |  |  |  |
| AT3G24830 | Ribosomal protein L13 family protein | ✔ |  |  |  |
| AT3G60680 | Plant protein of unknown function (DUF641) | ✔ | ✕ |  |  |
| AT2G37270 | RIBOSOMAL PROTEIN 5B (RPS5B) | ✔ |  |  |  |
| AT1G17260 | AUTOINHIBITED H(+)-ATPASE ISOFORM 10 (AHA10) | ✔ |  |  |  |
| AT4G27440 | PROTOCHLOROPHYLLIDE OXIDOREDUCTASE B | ✔ |  |  |  |
| AT5G28540 | Heat shock protein 70 BIP1 | ✔ |  | ✕ |  |
| AT1G79930 | HEAT SHOCK PROTEIN 91 (HSP91) | ✔ |  |  |  |
| ATCG00800 | RESISTANCE TO PSEUDOMONAS SYRINGAE 3 | ✔ |  |  |  |
| AT5G19990 | REGULATORY PARTICLE TRIPLE-A ATPASE 6A | ✔ |  |  |  |
| AT1G08360 | Ribosomal protein L1p/L10e family | ✔ |  |  |  |
| AT2G27530 | Ribosomal protein L10aP, PIGGYBACK1 (PGY1) | ✔ |  |  |  |
| AT1G02780 | EMBRYO DEFECTIVE 2386 (emb2386) | ✔ |  |  |  |
| AT5G41520 | RNA binding Plectin/S10 domain-containing protein | ✔ |  |  |  |
| AT4G39980 | 3-DEOXY-D-ARABINO-HEPTULOSONATE 7-PHOSPHATE SYNTHASE 1 (DHS1) | ✔ |  |  |  |
| AT1G22410 | Class-II DAHP synthetase family protein | ✔ |  |  |  |
| AT4G16720 | Ribosomal protein L23/L15e family protein | ✔ |  |  |  |
| AT1G71820 | Exocyst complex gene family, SEC6 | ✔ |  |  |  |
| AT1G29300 | Plant protein of unknown function (DUF641), UNFERTILIZED EMBRYO SAC 1 (UNE1) | ✔ | ✔ | ✔ | ✔ |
| AT4G15000 | Ribosomal L27e protein family | ✔ |  |  |  |
| AT3G56150 | EUKARYOTIC TRANSLATION INITIATION FACTOR 3C | ✔ |  |  |  |
| AT2G32730 | 26S proteasome regulatory complex, non-ATPase subcomplex, Rpn2/Psmd1 subunit | ✔ |  |  |  |
| AT1G04810 | 26S proteasome regulatory complex, non-ATPase subcomplex, Rpn2/Psmd1 subunit | ✔ |  |  |  |
| AT1G29930 | CHLOROPHYLL A/B BINDING PROTEIN 1 (CAB1) | ✔ |  |  |  |
| AT4G01400 | Unknown protein | ✔ |  |  |  |
| AT4G10340 | LIGHT HARVESTING COMPLEX OF PHOTOSYSTEM II 5 (LHCB5) | ✔ |  |  |  |
| AT2G33800 | Ribosomal protein S5 family protein, EMBRYO DEFECTIVE 3113 (EMB3113) | ✔ |  |  |  |
| AT3G54110 | PLANT UNCOUPLING MITOCHONDRIAL PROTEIN 1 (PUMP1) | ✔ |  |  |  |
| AT1G26880 | Ribosomal protein L34e superfamily protein | ✔ |  |  |  |
| AT3G45030 | Ribosomal protein S10p/S20e family protein | ✔ |  |  |  |
| AT3G47370 | Ribosomal protein S10p/S20e family protein | ✔ |  |  |  |
| AT5G19820 | EMBRYO DEFECTIVE 2734 (emb2734) | ✔ |  |  |  |
| AT3G02780 | ISOPENTENYL PYROPHOSPHATE:DIMETHYLALLYL PYROPHOSPHATE ISOMERASE 2 (IPP2) | ✔ |  |  |  |
| AT1G07770 | RIBOSOMAL PROTEIN S15A (RPS15A) | ✔ |  |  |  |
| AT3G47470 | LIGHT-HARVESTING CHLOROPHYLL-PROTEIN COMPLEX I SUBUNIT A4 (LHCA4) | ✔ |  |  |  |
| AT4G26740 | SEED GENE 1 (ATS1) | ✔ |  |  |  |
| AT3G53430 | Ribosomal protein L11 family protein | ✔ |  |  |  |
| AT2G37190 | Ribosomal protein L11 family protein | ✔ |  |  |  |
| AT3G07300 | NagB/RpiA/CoA transferase-like superfamily protein | ✔ |  |  |  |
| AT2G39390 | Ribosomal L29 family protein | ✔ |  |  |  |
| AT2G44060 | Late embryogenesis abundant protein, group 2 | ✔ |  |  |  |
| AT3G55280 | 60S ribosomal protein L23A (RPL23aB) | ✔ |  |  |  |
| AT1G70770 | Protein of unknown function DUF2359 | ✔ |  |  |  |
| AT4G35090 | CATALASE 2 (CAT2) | ✔ |  |  |  |
| AT1G20620 | CATALASE 3 (CAT3) | ✔ |  |  |  |
| AT1G14830 | DYNAMIN-LIKE 1C (DL1C) | ✔ |  |  |  |
| AT5G47190 | Ribosomal protein L19 family protein | ✔ |  |  |  |
| AT4G32410 | CELLULOSE SYNTHASE 1 (CESA1) | ✔ |  |  |  |
| AT1G48830 | Ribosomal protein S7e family protein | ✔ |  |  |  |
| AT1G76010 | Alba DNA/RNA-binding protein | ✔ | ✕ |  |  |
| AT4G33650 | DYNAMIN-RELATED PROTEIN 3A (DRP3A) | ✔ |  |  |  |
| AT5G52650 | RNA binding Plectin/S10 domain-containing protein | ✔ |  |  |  |
| AT5G23540 | Mov34/MPN/PAD-1 family protein | ✔ |  |  |  |
| AT4G20850 | TRIPEPTIDYL PEPTIDASE II (TPP2) | ✔ |  |  |  |
| AT5G10360 | EMBRYO DEFECTIVE 3010 (EMB3010) | ✔ |  |  |  |
| AT4G38190 | CELLULOSE SYNTHASE LIKE D4 (CSLD4) | ✔ |  |  |  |
| AT1G72340 | NagB/RpiA/CoA transferase-like superfamily protein | ✔ |  |  |  |
| AT2G46520 | cellular apoptosis susceptibility protein, putative / importin-alpha re-exporter, putative | ✔ |  |  |  |
| AT5G38640 | NagB/RpiA/CoA transferase-like superfamily protein | ✔ |  |  |  |
| AT1G74050 | Ribosomal protein L6 family protein | ✔ |  |  |  |
| AT5G05780 | Encodes a putative 26S proteasome subunit RPN8a | ✔ |  |  |  |
| AT3G54210 | Ribosomal protein L17 family protein | ✔ |  |  |  |
| AT2G38750 | ANNEXIN 4 (ANNAT4) | ✔ |  |  |  |
| AT5G20010 | RAS-RELATED NUCLEAR PROTEIN-1 (RAN-1) | ✔ |  |  |  |
| ATCG00770 | Chloroplast 30S ribosomal protein S8 | ✔ |  |  |  |
| AT5G65110 | ACYL-COA OXIDASE 2 (ACX2) | ✔ |  |  |  |
| AT2G10940 | Bifunctional inhibitor/lipid-transfer protein/seed storage 2S albumin superfamily protein | ✔ |  |  |  |
| AT4G24820 | 26S proteasome, regulatory subunit Rpn7 | ✔ |  |  |  |
| AT1G31330 | PHOTOSYSTEM I SUBUNIT F (PSAF) | ✔ |  |  |  |
| AT2G43030 | Ribosomal protein L3 family protein | ✔ |  |  |  |
| ATCG00160 | Chloroplast ribosomal protein S2 | ✔ |  |  |  |
| AT5G05170 | CONSTITUTIVE EXPRESSION OF VSP 1 (CEV1) | ✔ |  |  |  |
| AT2G47650 | UDP-XYLOSE SYNTHASE 4 (UXS4) | ✔ |  |  |  |
| AT5G42080 | DYNAMIN-LIKE PROTEIN (DL1) | ✔ |  |  |  |
| AT5G20630 | GERMIN 3 (GER3) | ✔ |  |  |  |
| AT5G45620 | Proteasome component (PCI) domain protein | ✔ |  |  |  |
| AT4G39200 | Ribosomal protein S25 family protein | ✔ |  |  |  |
| AT1G64520 | Regulatory particle non-ATPase 12A (RPN12a) | ✔ |  |  |  |
| AT3G07770 | HEAT SHOCK PROTEIN 89.1 (Hsp89.1) | ✔ |  |  |  |
| AT4G17530 | RAB GTPASE HOMOLOG 1C (RAB1C) | ✔ |  |  |  |
| AT1G15930 | Ribosomal protein L7Ae/L30e/S12e/Gadd45 family protein | ✔ |  |  |  |
| AT2G32060 | Ribosomal protein L7Ae/L30e/S12e/Gadd45 family protein | ✔ |  |  |  |
| AT1G12920 | EUKARYOTIC RELEASE FACTOR 1-2 (ERF1-2) | ✔ |  |  |  |
| AT3G22640 | PAP85 | ✔ |  |  |  |
| AT4G34870 | ROTAMASE CYCLOPHILIN 5 (ROC5) | ✔ |  |  |  |
| AT1G56330 | SECRETION-ASSOCIATED RAS 1B (SAR1B) | ✔ |  |  |  |
| AT3G56940 | COPPER RESPONSE DEFECT 1 (CRD1) | ✔ |  |  |  |
| AT5G09900 | EMBRYO DEFECTIVE 2107 (EMB2107) | ✔ |  |  |  |
| AT4G25630 | FIBRILLARIN 2 (FIB2) | ✔ |  |  |  |
| AT2G39730 | RUBISCO ACTIVASE (RCA) | ✔ |  |  |  |
| AT4G01100 | ADENINE NUCLEOTIDE TRANSPORTER 1 (ADNT1) | ✔ |  |  |  |
| AT3G19760 | DEAD-box RNA-helicase (EIF4A-III) | ✔ |  |  |  |
| ATCG00750 | 30S chloroplast ribosomal protein S11 | ✔ |  |  |  |
| AT5G11420 | Protein of unknown function | ✔ |  |  |  |
| AT1G67090 | RIBULOSE BISPHOSPHATE CARBOXYLASE SMALL CHAIN 1A (RBCS1A) | ✔ |  |  |  |
| AT3G09790 | UBIQUITIN 8 (UBQ8) | ✔ |  |  |  |
| AT3G14870 | Plant protein of unknown function (DUF641) | ✔ | ✕ |  |  |
| AT1G08730 | Class XI myosin gene | ✔ |  |  |  |
| AT3G60245 | Zinc-binding ribosomal protein family protein | ✔ |  |  |  |
| AT1G69620 | Putative 60S ribosomal protein L34 | ✔ |  |  |  |
| AT1G53880 | Eukaryotic translation initiation factor 2B (eIF-2B) family protein | ✔ |  |  |  |
| AT5G23740 | Encodes a putative ribosomal protein S11 (RPS11-beta) | ✔ |  |  |  |
| AT5G56010 | HEAT SHOCK PROTEIN 81-3 (HSP81-3) | ✔ |  |  |  |
| AT1G36160 | ACETYL-COA CARBOXYLASE 1 (ACC1) | ✔ |  |  |  |
| AT3G53990 | Adenine nucleotide alpha hydrolases-like superfamily protein | ✔ |  |  |  |
| AT2G13930 | Transposable element gene | ✔ |  |  |  |
| AT3G46040 | RIBOSOMAL PROTEIN S15A D (RPS15AD) | ✔ |  |  |  |
| AT1G36180 | ACETYL-COA CARBOXYLASE 2 (ACC2) | ✔ |  |  |  |
| AT5G64740 | CELLULOSE SYNTHASE 6 (CESA6) | ✔ |  |  |  |
| AT1G67520 | lectin protein kinase family protein | ✔ |  |  |  |
| AT3G23890 | TOPOISOMERASE II (TOPII) | ✔ |  |  |  |
| AT3G01460 | METHYL-CPG-BINDING DOMAIN 9 (MBD9) | ✔ |  |  |  |
| AT5G44800 | CHROMATIN REMODELING 4 (CHR4) | ✔ |  |  |  |
| AT5G45050 | WRKY Transcription Factor (WRKY16) | ✔ | ✕ | ✕ |  |
| AT5G44330 | Tetratricopeptide repeat (TPR)-like superfamily protein | ✔ |  |  |  |
| AT3G22230 | Ribosomal L27e protein family | ✔ |  |  |  |
| AT1G32120 | Protein of unknown function | ✔ |  |  |  |
| AT2G14120 | DYNAMIN RELATED PROTEIN (DRP3B) | ✔ |  |  |  |
| AT5G20490 | Encodes a member of the type XI myosin protein family | ✔ |  |  |  |
| AT1G01960 | EMBRYO SAC DEVELOPMENT ARREST 10 (EDA10) | ✔ |  |  |  |
| AT3G16550 | DEGP PROTEASE 12 (DEGP12) | ✔ |  |  |  |
| AT1G51060 | HISTONE H2A 10 (HTA10) | ✔ |  |  |  |
| AT3G48770 | DNA binding;ATP binding protein | ✔ |  |  |  |
| AT5G61340 | Protein of unknown function | ✔ |  |  |  |
| AT5G24740 | Protein of unknown function | ✔ |  |  |  |
| AT1G34300 | lectin protein kinase family protein | ✔ |  |  |  |
| AT5G50420 | O-fucosyltransferase family protein | ✔ |  |  |  |
| AT1G03060 | Encodes a WD/BEACH domain protein involved in cell morphogenesis. | ✔ |  |  |  |
| AT5G16910 | CELLULOSE-SYNTHASE LIKE D2 (CSLD2) | ✔ |  |  |  |
| AT3G48190 | ATAXIA-TELANGIECTASIA MUTATED (ATM) | ✔ |  |  |  |
| AT4G31900 | PICKLE RELATED 2 (PKR2) | ✔ |  |  |  |
| AT2G23890 | HAD-superfamily hydrolase, subfamily IG, 5'-nucleotidase | ✔ |  |  |  |
| AT3G19050 | PHRAGMOPLAST ORIENTING KINESIN 2 (POK2) | ✔ |  |  |  |
| AT1G19835 | Plant protein of unknown function (DUF869 | ✔ |  |  |  |
| AT5G35170 | adenylate kinase family protein | ✔ |  |  |  |
| AT1G03630 | PROTOCHLOROPHYLLIDE OXIDOREDUCTASE C (POR C) | ✔ |  |  |  |
| AT4G36630 | EMBRYO DEFECTIVE 2754 (EMB2754) | ✔ |  |  |  |
| AT4G25740 | RNA binding Plectin/S10 domain-containing protein | ✔ |  |  |  |
| ATCG00860 | Protein of unknown function | ✔ |  |  |  |
| AT3G22790 | Encodes a member of the NET superfamily of proteins | ✔ |  |  |  |
| AT3G09900 | RAB GTPase homolog E1E (RABE1e) | ✔ |  |  |  |
| AT5G42030 | ABL INTERACTOR-LIKE PROTEIN 4 (ABIL4) | ✔ |  |  |  |
| AT3G23690 | basic helix-loop-helix (bHLH77) DNA-binding superfamily protein | ✔ |  | ✕ | ✕ |
| AT4G28650 | Leucine-rich repeat transmembrane protein kinase family protein | ✔ |  |  |  |
| AT5G55340 | MBOAT (membrane bound O-acyl transferase) family protein | ✔ |  |  |  |
| AT2G33100 | CELLULOSE SYNTHASE-LIKE D1 (CSLD1) | ✔ |  |  |  |
| AT5G45210 | Disease resistance protein (TIR-NBS-LRR class) family | ✔ |  |  |  |
| AT3G57060 | Condensin complex subunit | ✔ |  |  |  |
| AT2G42100 | Actin-like ATPase superfamily protein | ✔ |  |  |  |
| AT4G18130 | Histidine Kinase | ✔ |  |  |  |
| AT4G18465 | RNA helicase family protein | ✔ |  |  |  |
| AT4G14760 | kinase interacting (KIP1-like) family protein | ✔ |  |  |  |
| AT1G40390 | DNAse I-like superfamily protein | ✔ |  |  |  |
| AT5G16440 | ISOPENTENYL DIPHOSPHATE ISOMERASE 1 (IPP1) | ✔ |  |  |  |
| AT2G33240 | MYOSIN XI D (XID) | ✔ |  |  |  |
| AT2G05070 | PHOTOSYSTEM II LIGHT HARVESTING COMPLEX GENE 2.2 (LHCB2.2) | ✔ |  |  |  |
| AT5G47200 | RAB GTPASE HOMOLOG 1A (RAB1A) | ✔ |  |  |  |
| AT1G02130 | RAS 5 (RA-5) | ✔ |  |  |  |
| AT3G02260 | Calossin-like protein BIG | ✔ |  |  |  |
| AT3G59100 | GLUCAN SYNTHASE-LIKE 11 (GSL11) | ✔ |  |  |  |
| AT5G49310 | IMPORTIN ALPHA ISOFORM 5 (IMPA-5) | ✔ |  |  |  |
| AT5G65630 | GLOBAL TRANSCRIPTION FACTOR GROUP E7 (GTE7) | ✔ | ✕ |  |  |
| AT3G50240 | Encodes a kinesin-related protein. | ✔ |  |  |  |
| AT5G23390 | Plant protein of unknown function (DUF639) | ✔ |  |  |  |
| AT1G18040 | CYCLIN-DEPENDENT KINASE D1;3 (CDKD1;3) | ✔ | ✕ |  |  |
| AT4G34530 | CRYPTOCHROME-INTERACTING BASIC-HELIX-LOOP-HELIX 1 (CIB1, bHLH63) | ✔ |  |  |  |
| AT3G16390 | NITRILE SPECIFIER PROTEIN 3 (NSP3) | ✔ |  |  |  |
| AT5G62070 | IQ-DOMAIN 23 (IQD23) | ✔ |  |  |  |
| AT3G60240 | EUKARYOTIC TRANSLATION INITIATION FACTOR 4G (EIF4G) | ✔ |  |  |  |
| AT3G21380 | Mannose-binding lectin superfamily protein | ✔ |  |  |  |
| AT4G23640 | Potassium transporter TINY ROOT HAIR 1 (TRH1) | ✔ |  |  |  |
| AT5G51230 | EMBRYONIC FLOWER 2 (EMF2) | ✔ | ✕ | ✕ |  |
| AT3G10060 | FKBP-like peptidyl-prolyl cis-trans isomerase family protein | ✔ |  |  |  |
| AT3G03050 | CELLULOSE SYNTHASE-LIKE D3 (CSLD3) | ✔ |  |  |  |
| AT5G44030 | CELLULOSE SYNTHASE A4 (CESA4) | ✔ |  |  |  |
| AT4G33630 | EXECUTER1 (EX1) | ✔ |  |  |  |
| AT4G34370 | ARIADNE 1 (ARI1) | ✔ |  |  |  |
| AT1G56410 | EARLY-RESPONSIVE TO DEHYDRATION 2 (ERD2) | ✔ |  |  |  |
| AT5G43900 | MYOSIN 2 (MYA2) | ✔ |  |  |  |
| AT5G64960 | CYCLIN DEPENDENT KINASE GROUP C2 (CDKC2) | ✔ |  |  |  |
| AT5G60670 | Ribosomal protein L11 family protein | ✔ |  |  |  |
| AT5G09870 | CELLULOSE SYNTHASE 5 (CESA5) | ✔ |  |  |  |
| AT1G04120 | ATP-BINDING CASSETTE C5 (ABCC5) | ✔ |  |  |  |
| AT4G14370 | Disease resistance protein (TIR-NBS-LRR class) family | ✔ |  |  |  |
| AT1G11410 | S-locus lectin protein kinase family protein | ✔ |  |  |  |
| AT1G66290 | F-box/RNI-like superfamily protein | ✔ |  |  |  |
| AT4G13170 | Ribosomal protein L13 family protein | ✔ |  |  |  |
| AT1G66320 | F-box/RNI-like superfamily protein | ✔ |  |  |  |
| AT5G65250 | Protein of unknown function | ✔ |  |  |  |
| AT1G09080 | BINDING PROTEIN 3 (BIP3) | ✔ |  |  |  |
| AT3G16780 | Ribosomal protein L19e family protein | ✔ |  |  |  |
| AT4G16650 | O-fucosyltransferase family protein | ✔ |  |  |  |
| AT4G39280 | phenylalanyl-tRNA synthetase, putative / phenylalanine--tRNA ligase, putative | ✔ |  |  |  |
| AT2G31810 | ACT domain-containing small subunit of acetolactate synthase protein | ✔ |  |  |  |
| AT4G36100 | Sec1/munc18-like (SM) proteins superfamily | ✔ |  |  |  |
| AT2G34050 | Protein of unknown function | ✔ |  |  |  |
| AT4G30790 | Protein of unknown function | ✔ |  |  |  |

Y2H: yeast two-hybrid.

BiFC: bimolecular fluorescence complementation.

✔: positive interaction

✕: negative interaction

blank: not tested

**Table S9.** All-by-all protein-protein interaction studies in yeast among nine CHIQ family members.

|  | AT1G29300 | AT2G32130 | AT3G14870 | AT1G53380 | AT4G34080 | AT2G45260 | AT4G33320 | AT5G60680 | AT5G58960 |
| --- | --- | --- | --- | --- | --- | --- | --- | --- | --- |
| AT1G29300 |  | ✕ | ✕ | ✕ | ✕ | ✔ | ✕ | ✕ | ✕ |
| AT2G32130 |  |  | ✕ | ✕ | ✕ | ✔ | ✕ | ✕ | ✕ |
| AT3G14870 |  |  |  | ✕ | ✕ | ✕ | ✕ | ✕ | ✕ |
| AT1G53380 |  |  |  |  | ✕ | ✔ | ✕ | ✕ | ✕ |
| AT4G34080 |  |  |  |  |  | ✕ | ✕ | ✕ | ✕ |
| AT2G45260 |  |  |  |  |  |  | ✕ | ✕ | ✕ |
| AT4G33320 |  |  |  |  |  |  |  | ✕ | ✕ |
| AT5G60680 |  |  |  |  |  |  |  |  | ✕ |
| AT5G58960 |  |  |  |  |  |  |  |  |  |

✔: positive interaction

✕: negative interaction

blank: not tested

**References**

1. Pagnussat GC, Yu H-J, Ngo QA, Rajani S, Mayalagu S, Johnson CS, Capron A, Xie L-F, Ye D, Sundaresan V. Genetic and molecular identification of genes required for female gametophyte development and function in Arabidopsis. Development. 2005;132(3):603-14.

2. Allen T, Ingles PJ, Praekelt U, Smith H, Whitelam GC. Phytochrome-mediated agravitropism in Arabidopsis hypocotyls requires GIL1 and confers a fitness advantage. Plant J. 2006;46(4):641-8.

3. Wilson-Sánchez D, Rubio-Díaz S, Muñoz-Viana R, Pérez-Pérez JM, Jover-Gil S, Ponce MR, Micol JL. Leaf phenomics: a systematic reverse genetic screen for Arabidopsis leaf mutants. Plant J. 2014;79(5):878-91.

4. Iyer LM, Aravind L. ALOG domains: provenance of plant homeotic and developmental regulators from the DNA-binding domain of a novel class of DIRS1-type retroposons. Biol Direct. 2012;7:39.

5. Cho E, Zambryski PC. ORGAN BOUNDARY1 defines a gene expressed at the junction between the shoot apical meristem and lateral organs. Proc Natl Acad Sci USA. 2011;108(5):2154-9.

6. Takeda S, Hanano K, Kariya A, Shimizu S, Zhao L, Matsui M, Tasaka M, Aida M. CUP-SHAPED COTYLEDON1 transcription factor activates the expression of LSH4 and LSH3, two members of the ALOG gene family, in shoot organ boundary cells. Plant J. 2011;66(6):1066-77.

7. Luhua S, Hegie A, Suzuki N, Shulaev E, Luo X, Cenariu D, Ma V, Kao S, Lim J, Gunay MB et al. Linking genes of unknown function with abiotic stress responses by high-throughput phenotype screening. Physiol Plant. 2013;148(3):322-33.

8. Zhao L, Nakazawa M, Takase T, Manabe K, Kobayashi M, Seki M, Shinozaki K, Matsui M. Overexpression of LSH1, a member of an uncharacterised gene family, causes enhanced light regulation of seedling development. Plant J. 2004;37(5):694-706.

9. Husbands A, Bell EM, Shuai B, Smith HMS, Springer PS. LATERAL ORGAN BOUNDARIES defines a new family of DNA-binding transcription factors and can interact with specific bHLH proteins. Nucleic Acids Res. 2007;35(19):6663-71.

10. Rubin G, Tohge T, Matsuda F, Saito K, Scheible W-R. Members of the LBD Family of Transcription Factors Repress Anthocyanin Synthesis and Affect Additional Nitrogen Responses in Arabidopsis. Plant Cell. 2009;21(11):3567-84.

11. Pecher P, Eschen-Lippold L, Herklotz S, Kuhle K, Naumann K, Bethke G, Uhrig J, Weyhe M, Scheel D, Lee J. The Arabidopsis thaliana mitogen-activated protein kinases MPK3 and MPK6 target a subclass of ‘VQ-motif’-containing proteins to regulate immune responses. New Phytol. 2014;203(2):592-606.

12. Yang Y, Sage TL, Liu Y, Ahmad TR, Marshall WF, Shiu S-H, Froehlich JE, Imre KM, Osteryoung KW. CLUMPED CHLOROPLASTS 1 is required for plastid separation in Arabidopsis. Proc Natl Acad Sci USA. 2011;108(45):18530-5.

13. Wang A, Garcia D, Zhang H, Feng K, Chaudhury A, Berger F, Peacock WJ, Dennis ES, Luo M. The VQ motif protein IKU1 regulates endosperm growth and seed size in Arabidopsis. Plant J. 2010;63(4):670-9.

14. Garcia D, Saingery V, Chambrier P, Mayer U, Jürgens G, Berger F. Arabidopsis haiku Mutants Reveal New Controls of Seed Size by Endosperm. Plant Physiol. 2003;131(4):1661-70.

15. Cheng Y, Zhou Y, Yang Y, Chi Y-J, Zhou J, Chen J-Y, Wang F, Fan B, Shi K, Zhou Y-H et al. Structural and Functional Analysis of VQ Motif-Containing Proteins in Arabidopsis as Interacting Proteins of WRKY Transcription Factors. Plant Physiol. 2012;159(2):810-25.

16. Kogan GL, Gvozdev VA. Multifunctional nascent polypeptide-associated complex (NAC). Mol Biol. 2014;48(2):189-96.

17. Varier RA, Carrillo de Santa Pau E, van der Groep P, Lindeboom RG, Matarese F, Mensinga A, Smits AH, Edupuganti RR, Baltissen MP, Jansen PW et al. Recruitment of the Mammalian Histone-modifying EMSY Complex to Target Genes Is Regulated by ZNF131. J Biol Chem. 2016;291(14):7313-24.

18. Mulrane L, Gallagher WM, O'Connor DP. A novel mechanism of regulation of the anti-metastatic miR-31 by EMSY in breast cancer. Breast Cancer Res. 2014;16(6):467.

19. Garapaty S, Xu CF, Trojer P, Mahajan MA, Neubert TA, Samuels HH. Identification and characterization of a novel nuclear protein complex involved in nuclear hormone receptor-mediated gene regulation. J Biol Chem. 2009;284(12):7542-52.

20. Hughes-Davies L, Huntsman D, Ruas M, Fuks F, Bye J, Chin SF, Milner J, Brown LA, Hsu F, Gilks B et al. EMSY links the BRCA2 pathway to sporadic breast and ovarian cancer. Cell. 2003;115(5):523-35.

21. Vermeulen M, Eberl HC, Matarese F, Marks H, Denissov S, Butter F, Lee KK, Olsen JV, Hyman AA, Stunnenberg HG et al. Quantitative interaction proteomics and genome-wide profiling of epigenetic histone marks and their readers. Cell. 2010;142(6):967-80.

22. Vire E, Curtis C, Davalos V, Git A, Robson S, Villanueva A, Vidal A, Barbieri I, Aparicio S, Esteller M et al. The breast cancer oncogene EMSY represses transcription of antimetastatic microRNA miR-31. Mol Cell. 2014;53(5):806-18.

23. Tsuchiya T, Eulgem T. EMSY-Like Genes Are Required for Full RPP7-Mediated Race-Specific Immunity and Basal Defense in Arabidopsis. Mol Plant Microbe Interact. 2011;24(12):1573-81.

24. Wang S, Chang Y, Guo J, Zeng Q, Ellis BE, Chen J-G. Arabidopsis Ovate Family Proteins, a Novel Transcriptional Repressor Family, Control Multiple Aspects of Plant Growth and Development. PloS one. 2011;6(8):e23896.

25. Dean PJ, Siwiec T, Waterworth WM, Schlögelhofer P, Armstrong SJ, West CE. A novel ATM-dependent X-ray-inducible gene is essential for both plant meiosis and gametogenesis. Plant J. 2009;58(5):791-802.

26. Petricka JJ, Clay NK, Nelson TM. Vein patterning screens and the defectively organized tributaries mutants in Arabidopsis thaliana. Plant J. 2008;56(2):251-63.

27. Masaki T, Tsukagoshi H, Mitsui N, Nishii T, Hattori T, Morikami A, Nakamura K. Activation tagging of a gene for a protein with novel class of CCT-domain activates expression of a subset of sugar-inducible genes in Arabidopsis thaliana. Plant J. 2005;43(1):142-52.

28. Perez-Rodriguez P, Riano-Pachon DM, Correa LG, Rensing SA, Kersten B, Mueller-Roeber B. PlnTFDB: updated content and new features of the plant transcription factor database. Nucleic Acids Res. 2010;38:D822-7.

29. Curaba J, Herzog M, Vachon G. GeBP, the first member of a new gene family in Arabidopsis, encodes a nuclear protein with DNA-binding activity and is regulated by KNAT1. Plant J. 2003;33(2):305-17.

30. Chevalier F, Perazza D, Laporte F, Le Henanff G, Hornitschek P, Bonneville JM, Herzog M, Vachon G. GeBP and GeBP-like proteins are noncanonical leucine-zipper transcription factors that regulate cytokinin response in Arabidopsis. Plant Physiol. 2008;146(3):1142-54.

31. Neumuller RA, Richter C, Fischer A, Novatchkova M, Neumuller KG, Knoblich JA. Genome-wide analysis of self-renewal in Drosophila neural stem cells by transgenic RNAi. Cell Stem Cell. 2011;8(5):580-93.

32. Mummery-Widmer JL, Yamazaki M, Stoeger T, Novatchkova M, Bhalerao S, Chen D, Dietzl G, Dickson BJ, Knoblich JA. Genome-wide analysis of Notch signalling in Drosophila by transgenic RNAi. Nature. 2009;458(7241):987-92.

33. Mohan M, Herz HM, Smith ER, Zhang Y, Jackson J, Washburn MP, Florens L, Eissenberg JC, Shilatifard A. The COMPASS Family of H3K4 Methylases in Drosophila. Mol Cel Biol. 2011;31(21):4310-8.

34. Vardanyan A, Atanesyan L, Egli D, Raja SJ, Steinmann-Zwicky M, Renkawitz-Pohl R, Georgiev O, Schaffner W. Dumpy-30 family members as determinants of male fertility and interaction partners of metal-responsive transcription factor 1 (MTF-1) in Drosophila. BMC Dev Biol. 2008;8:68.

35. Bhaskar V, Courey AJ. The MADF-BESS domain factor Dip3 potentiates synergistic activation by Dorsal and Twist. Gene. 2002;299(1-2):173-84.

36. Carreira VP, Soto IM, Mensch J, Fanara JJ. Genetic basis of wing morphogenesis in Drosophila: sexual dimorphism and non-allometric effects of shape variation. BMC Dev Biol. 2011;11:32.

37. Edwards AC, Zwarts L, Yamamoto A, Callaerts P, Mackay TF. Mutations in many genes affect aggressive behavior in Drosophila melanogaster. BMC Biol. 2009;7:29.

38. Guest ST, Yu J, Liu D, Hines JA, Kashat MA, Finley RL. A protein network-guided screen for cell cycle regulators in Drosophila. BMC Syst Biol. 2011;5(1):65.

39. Vandepoele K, Van Roy N, Staes K, Speleman F, van Roy F. A Novel Gene Family NBPF: Intricate Structure Generated by Gene Duplications During Primate Evolution. Mol Biol Evol. 2005;22(11):2265-74.

40. Li Y, Dong X, Yin Y, Su Y, Xu Q, Zhang Y, Pang X, Zhang Y, Chen W. BJ-TSA-9, a Novel Human Tumor-Specific Gene, Has Potential as a Biomarker of Lung Cancer. Neoplasia. 2005;7(12):1073-80.

41. Kim JW, Lee SK, Lee ZH, Park JC, Lee KE, Lee MH, Park JT, Seo BM, Hu JC, Simmer JP. FAM83H mutations in families with autosomal-dominant hypocalcified amelogenesis imperfecta. Am J Hum Genet. 2008;82(2):489-94.

42. Vogt J, Dingwell KS, Herhaus L, Gourlay R, Macartney T, Campbell D, Smith JC, Sapkota GP. Protein associated with SMAD1 (PAWS1/FAM83G) is a substrate for type I bone morphogenetic protein receptors and modulates bone morphogenetic protein signalling. Open Biol. 2014;4:130210.

43. Massagué J, Blain SW, Lo RS. TGFβ Signaling in Growth Control, Cancer, and Heritable Disorders. Cell. 2000;103(2):295-309.

44. Davidson G, Murphy S, Polke J, Laura M, Salih M, Muntoni F, Blake J, Brandner S, Davies N, Horvath R et al. Frequency of mutations in the genes associated with hereditary sensory and autonomic neuropathy in a UK cohort. J Neurol. 2012;259(8):1673-85.

45. Kurth I, Pamminger T, Hennings JC, Soehendra D, Huebner AK, Rotthier A, Baets J, Senderek J, Topaloglu H, Farrell SA et al. Mutations in FAM134B, encoding a newly identified Golgi protein, cause severe sensory and autonomic neuropathy. Nat Genet. 2009;41(11):1179-81.

46. Lai F, Godley LA, Fernald AA, Orelli BJ, Pamintuan L, Zhao N, Le Beau MM. cDNA cloning and genomic structure of three genes localized to human chromosome band 5q31 encoding potential nuclear proteins. Genomics. 2000;70(1):123-30.

47. Marshall D, Hardman MJ, Nield KM, Byrne C. Differentially expressed late constituents of the epidermal cornified envelope. Proc Natl Acad Sci USA. 2001;98(23):13031-6.

48. Jackson B, Tilli CM, Hardman MJ, Avilion AA, MacLeod MC, Ashcroft GS, Byrne C. Late cornified envelope family in differentiating epithelia-response to calcium and ultraviolet irradiation. J Invest Dermatol. 2005;124(5):1062-70.

49. Kurima K, Yang Y, Sorber K, Griffith AJ. Characterization of the transmembrane channel-like (TMC) gene family: functional clues from hearing loss and epidermodysplasia verruciformis. Genomics. 2003;82(3):300-8.

50. Pan B, Géléoc Gwenaelle S, Asai Y, Horwitz Geoffrey C, Kurima K, Ishikawa K, Kawashima Y, Griffith Andrew J, Holt Jeffrey R. TMC1 and TMC2 Are Components of the Mechanotransduction Channel in Hair Cells of the Mammalian Inner Ear. Neuron. 2013;79(3):504-15.

51. Dortay H, Gruhn N, Pfeifer A, Schwerdtner M, Schmulling T, Heyl A. Toward an interaction map of the two-component signaling pathway of Arabidopsis thaliana. J Proteome Res. 2008;7(9):3649-60.

52. Kim MH, Sonoda Y, Sasaki K, Kaminaka H, Imai R. Interactome analysis reveals versatile functions of Arabidopsis COLD SHOCK DOMAIN PROTEIN 3 in RNA processing within the nucleus and cytoplasm. Cell Stress Chaperones. 2013;18(4):517-25.

53. Causier B, Ashworth M, Guo W, Davies B. The TOPLESS interactome: a framework for gene repression in Arabidopsis. Plant Physiol. 2012;158(1):423-38.

54. Consortium AIM. Evidence for network evolution in an Arabidopsis interactome map. Science. 2011;333(6042):601-7.

55. Hu Y, Chen L, Wang H, Zhang L, Wang F, Yu D. Arabidopsis transcription factor WRKY8 functions antagonistically with its interacting partner VQ9 to modulate salinity stress tolerance. Plant J. 2013;74(5):730-45.

56. Pi L, Aichinger E, van der Graaff E, Llavata-Peris CI, Weijers D, Hennig L, Groot E, Laux T. Organizer-Derived WOX5 Signal Maintains Root Columella Stem Cells through Chromatin-Mediated Repression of CDF4 Expression. Dev Cell. 2015;33(5):576-88.

57. Hackbusch J, Richter K, Muller J, Salamini F, Uhrig JF. A central role of Arabidopsis thaliana ovate family proteins in networking and subcellular localization of 3-aa loop extension homeodomain proteins. Proc Natl Acad Sci USA. 2005;102(13):4908-12.

58. Servet C, Benhamed M, Latrasse D, Kim W, Delarue M, Zhou DX. Characterization of a phosphatase 2C protein as an interacting partner of the histone acetyltransferase GCN5 in Arabidopsis. Biochim Biophys Acta. 2008;1779(6-7):376-82.

59. Efroni I, Han S-K, Kim Hye J, Wu M-F, Steiner E, Birnbaum Kenneth D, Hong Jong C, Eshed Y, Wagner D. Regulation of Leaf Maturation by Chromatin-Mediated Modulation of Cytokinin Responses. Dev Cell. 2013;24(4):438-45.

60. Waidmann S, Kusenda B, Mayerhofer J, Mechtler K, Jonak C. A DEK domain-containing protein modulates chromatin structure and function in Arabidopsis. Plant Cell. 2014;26(11):4328-44.

61. Giot L, Bader JS, Brouwer C, Chaudhuri A, Kuang B, Li Y, Hao YL, Ooi CE, Godwin B, Vitols E et al. A protein interaction map of Drosophila melanogaster. Science. 2003;302(5651):1727-36.

62. Lunardi A, Di Minin G, Provero P, Dal Ferro M, Carotti M, Del Sal G, Collavin L. A genome-scale protein interaction profile of Drosophila p53 uncovers additional nodes of the human p53 network. Proc Natl Acad Sci USA. 2010;107(14):6322-7.

63. Guruharsha KG, Rual JF, Zhai B, Mintseris J, Vaidya P, Vaidya N, Beekman C, Wong C, Rhee DY, Cenaj O et al. A protein complex network of Drosophila melanogaster. Cell. 2011;147(3):690-703.

64. Vandepoele K, Staes K, Andries V, van Roy F. Chibby interacts with NBPF1 and clusterin, two candidate tumor suppressors linked to neuroblastoma. Exp Cell Res. 2010;316(7):1225-33.

65. Weimann M, Grossmann A, Woodsmith J, Ozkan Z, Birth P, Meierhofer D, Benlasfer N, Valovka T, Timmermann B, Wanker EE et al. A Y2H-seq approach defines the human protein methyltransferase interactome. Nat Methods. 2013;10(4):339-42.

66. Rual J-F, Venkatesan K, Hao T, Hirozane-Kishikawa T, Dricot A, Li N, Berriz GF, Gibbons FD, Dreze M, Ayivi-Guedehoussou N et al. Towards a proteome-scale map of the human protein-protein interaction network. Nature. 2005;437(7062):1173-8.

67. Chatraryamontri A, Breitkreutz B-J, Oughtred R, Boucher L, Heinicke S, Chen D, Stark C, Breitkreutz A, Kolas N, O'Donnell L et al. The BioGRID interaction database: 2015 update. Nucleic Acids Res. 2015;43(D1):D470-8.

68. Huttlin EL, Ting L, Bruckner RJ, Gebreab F, Gygi MP, Szpyt J, Tam S, Zarraga G, Colby G, Baltier K et al. The BioPlex Network: A Systematic Exploration of the Human Interactome. Cell. 2015;162(2):425-40.

69. Brown KA, Ham AJ, Clark CN, Meller N, Law BK, Chytil A, Cheng N, Pietenpol JA, Moses HL. Identification of novel Smad2 and Smad3 associated proteins in response to TGF-beta1. J Cell Biochem. 2008;105(2):596-611.

70. Rolland T, Tasan M, Charloteaux B, Pevzner SJ, Zhong Q, Sahni N, Yi S, Lemmens I, Fontanillo C, Mosca R et al. A proteome-scale map of the human interactome network. Cell. 2014;159(5):1212-26.
